# The Effect of Graft Type on Vertical Jump Performance After Anterior Cruciate Ligament Reconstruction: A Systematic Review

**DOI:** 10.3390/jcm15176588

**Published:** 2026-08-26

**Authors:** Krzysztof Kasicki, Grzegorz Błażejewski, Łukasz Rydzik, Piotr Wróbel, Monika Nowak, Tadeusz Ambroży

**Affiliations:** 1 Department of Sport Theory and Motor Skills, Institute of Sports Sciences, University of Physical Culture in Kraków, 31-571 Krakow, Poland; lukasz.rydzik@awf.krakow.pl (Ł.R.); tadeusz.ambrozy@awf.krakow.pl (T.A.); 2 Faculty of Health Sciences, Collegium Medicum, Andrzej Frycz Modrzewski Krakow University, 30-705 Krakow, Poland; gblazejewski@uafm.edu.pl (G.B.); pwrobel1@uafm.edu.pl (P.W.); mprzybytek@uafm.edu.pl (M.N.)

**Keywords:** ACLR, graft, countermovement jump, limb symmetry index, return to sport, vertical jump

## Abstract

**Background:** Anterior cruciate ligament reconstruction (ACLR) is the standard of care for ACL injuries, and graft choice may influence the recovery of function assessed with vertical jump tests. **Objective:** To evaluate the effect of graft type on vertical jump performance in adults after ACLR and to synthesise the differences between grafts. **Material and Methods:** This systematic review was conducted in accordance with the PRISMA 2020 and SWiM guidelines. The following databases were searched: Scopus, Web of Science, PubMed, MEDLINE and SPORTDiscus (EBSCOhost), up to 31 January 2026. Studies with an unambiguously defined graft type and objective jump tests (CMJ, SL CMJ/SL VJ, DVJ/DJ, SJ) were included. Risk of bias was assessed using the RoB 2, ROBINS-I, JBI and MINORS tools, and the certainty of evidence using the GRADE approach. Because of substantial clinical and methodological heterogeneity, a structured narrative synthesis was performed in accordance with the SWiM guidelines; no pooled analysis was undertaken. **Results:** Forty-seven studies (>3400 participants) were included. BPTB was associated with greater eccentric-phase asymmetry in the bilateral CMJ in the early/intermediate phase compared with HT, with equalisation after 9–12 months. QT provided jump height comparable to HT but a more favourable dynamic valgus profile. Autograft and allograft did not differ in jump performance. Deficits followed a biphasic course: normalisation of symmetry after approximately 2 years, with reduced height/power for up to 5 years. **Conclusions:** Forty-two of the studies (89.4%) were non-randomised, and the certainty of evidence was low for one clinical question and very low for all others. Studies comparing BPTB with HT showed an association suggesting greater eccentric-phase asymmetry in the bilateral CMJ in the early and intermediate phase after BPTB, but findings were inconsistent and two studies reported no difference. Evidence suggesting that these differences equalise after 9–12 months derives from between-study comparisons. Jump height did not differ between QT and HT; a single study of 26 patients reported smaller dynamic valgus after QT. Autograft and allograft did not differ in vertical jump performance. **Registration:** This review was registered in the PROSPERO database and is available under the number CRD420261386307.

## 1. Introduction

Injury of the anterior cruciate ligament (ACL) is a common injury among physically active individuals and athletes [1]. ACL reconstruction (ACLR) is a widely used surgical treatment method whose aim is to improve the function and stability of the knee joint and to enable return to physical activity and sport [2]. In clinical practice, several graft types are used, including patellar tendon autografts (BPTB), hamstring tendon autografts (HT), quadriceps tendon autografts (QT) and allografts. Differences related to the graft harvest site and to the course of postoperative adaptation may influence functional recovery after ACLR [3]. Assessment of functional recovery after ACLR is a key element of monitoring rehabilitation progress and of making decisions regarding return to sports activity [4]. Among the tools used in functional assessment after ACLR are vertical jump tests, which allow evaluation of the ability to generate and absorb force and to perform complex motor tasks. In contrast to simple measurements of muscle strength, jump tests provide information on the performance of complex dynamic tasks that better reflect the demands of sports activity [5]. Moreover, contemporary assessment methods allow analysis not only of jump height but also of other parameters, including, for example, inter-limb asymmetry, kinetic parameters, mechanical power and landing biomechanics [6]. The type of graft used may influence the course of functional recovery after ACLR because of the different biomechanical and functional properties resulting from the graft harvest site [7].

Studies indicate that the type of graft used may influence the way dynamic tasks are performed, which is reflected in differences in loading asymmetry and impulse asymmetry during the countermovement jump between patients after ACL reconstruction using BPTB and HT grafts [7,8].

Existing studies evaluating the effect of graft type on vertical jump performance differ with respect to the characteristics of the studied populations, the testing protocols used and the functional parameters analysed, which hampers direct comparison of results and the formulation of unequivocal clinical conclusions [7,9].

A systematic synthesis of the available evidence may contribute to a better understanding of the potential differences related to graft type and of their importance for functional assessment and rehabilitation planning after ACLR.

The general rationale for creating this systematic review was to assess the effect of the graft type used during anterior cruciate ligament reconstruction on the vertical jump parameters used to evaluate functional recovery after ACLR, and to synthesise the available evidence on differences between individual graft types.

### Objectives

The main aim of this systematic review was to assess the effect of the type of graft used in anterior cruciate ligament reconstruction (ACLR) on vertical jump performance in adult patients during the postoperative period. The specific objectives were defined as follows:

To compare vertical jump performance between the most commonly used autograft types, taking into account differences in jump height, mechanical power and limb symmetry indices;

To analyse differences in vertical jump performance between autografts and allografts;

To characterise the temporal course of vertical jump performance deficits from the early postoperative phase to long-term follow-up;

To formulate practical recommendations for clinicians and physiotherapists regarding the choice of rehabilitation strategy for a given graft type in the context of the expected vertical jump function, and to identify research gaps indicating directions for future randomised controlled trials.

## 2. Materials and Methods

This systematic review was conducted in accordance with the PRISMA (Preferred Reporting Items for Systematic Reviews and Meta-Analyses) and SWiM (Synthesis Without Meta-analysis in systematic reviews) guidelines [10]. The article was registered in the PROSPERO database and is publicly available under the number CRD420261386307. The PRISMA Checklist has been provided in the Supplementary Materials section as Table S1.

### 2.1. Literature Search Strategy and Sources

The search strategy consisted of identifying as many publications related to the topic as possible. Search queries were created for each database (Scopus, Web of Science, PubMed, MEDLINE, SPORTDiscus); the search phrases were selected so as to enable identification of articles related to anterior cruciate ligament reconstruction and to functional tests associated with vertical jump performance. PubMed and MEDLINE were searched as separate sources, because they differ in query syntax and the MeSH term mapping mechanism. The MEDLINE and SPORTDiscus databases were searched using EBSCOhost. The PubMed and MEDLINE databases overlap in terms of available records, but the overall goal was to minimise the risk of omitting literature eligible for this review. The only filters applied to each database were that the sources be peer-reviewed journals and that each text be available in English. The review was not restricted by year of publication. The review covered publications published up to 31 January 2026. Initially, without applying the two aforementioned filters, 1728 results were identified; after applying the filters, 1528 remained. The detailed search queries for each database are presented below:

- Scopus

TITLE-ABS-KEY ((“anterior cruciate ligament” OR “ACL”) AND (reconstruction OR “ACL reconstruction” OR “surgical reconstruction” OR graft* OR “patellar tendon graft” OR “hamstring tendon graft” OR “quadriceps tendon graft” OR autograft* OR allograft*) AND (“vertical jump” OR “jump performance” OR “jump height” OR “countermovement jump” OR CMJ OR “squat jump” OR SJ OR plyometric*))

- Web of Science

TS = ((“anterior cruciate ligament” OR ACL) AND (reconstruction OR “ACL reconstruction” OR graft* OR “patellar tendon” OR “hamstring tendon” OR “quadriceps tendon” OR autograft* OR allograft*) AND (“vertical jump” OR “jump performance” OR “jump height” OR “countermovement jump” OR CMJ OR “squat jump” OR SJ))

- PubMed

((“Anterior Cruciate Ligament Reconstruction”[Mesh] OR “Anterior Cruciate Ligament”[Mesh] OR ACL) AND (reconstruction OR graft* OR autograft* OR allograft* OR “patellar tendon” OR “hamstring tendon” OR “quadriceps tendon”) AND (“vertical jump” OR “jump performance” OR “jump height” OR “countermovement jump” OR CMJ OR “squat jump”))

- MEDLINE

((“Anterior Cruciate Ligament”[MeSH] OR “anterior cruciate ligament” OR ACL) AND (“Anterior Cruciate Ligament Reconstruction”[MeSH] OR “ACL reconstruction” OR reconstruction OR graft* OR autograft* OR allograft* OR “patellar tendon” OR “hamstring tendon” OR “quadriceps tendon”) AND (“vertical jump” OR “jump performance” OR “jump height” OR “countermovement jump” OR CMJ OR “squat jump” OR SJ))

- SPORTDiscus

((“anterior cruciate ligament” OR ACL) AND (“ACL reconstruction” OR reconstruction OR graft* OR autograft* OR allograft* OR “patellar tendon” OR “hamstring tendon” OR “quadriceps tendon”) AND (“vertical jump” OR “jump height” OR “jump performance” OR “countermovement jump” OR CMJ OR “squat jump” OR SJ OR plyometric*))

### 2.2. Eligibility Criteria (PICOS Framework)

The inclusion and exclusion criteria were established by the team on the basis of the PICOS criteria, in accordance with the recommendations of PRISMA and the Cochrane Handbook for Systematic Reviews of Interventions [10,11]. The detailed criteria are described in the following paragraphs.

With regard to the population, studies including adult patients aged ≥ 18 years, of both sexes, were eligible. Healthy individuals were admitted only as a control group. Animal studies, cadaveric studies, and publications including patients with bilateral ACL injury, concomitant reconstructions of other knee ligaments (PCL, MCL, LCL) or complex joint injuries were excluded.

The intervention comprised ACLR using a defined graft type: patellar tendon (BPTB), hamstring tendons (HT, ST, STGR), quadriceps tendon (QT), autograft or allograft. Unambiguous specification of the graft type used was required in the methods section of the included study; studies not specifying the graft, or reporting heterogeneous, non-stratified mixed groups, were excluded.

The comparison consisted of a direct comparison between different graft types, a comparison of the operated-limb results with the non-operated limb within the same patient using the limb symmetry index, or a comparison with a healthy control group. Studies without a classic comparison group were also accepted if they reported objective indices of inter-limb asymmetry or differences in functional outcomes.

The endpoints comprised objective measures of vertical jump performance obtained under laboratory or field conditions, including jump height; kinetic parameters such as ground reaction force, peak power, eccentric- and concentric-phase impulse and rate of force development; kinematic parameters, namely joint angles, joint moments and dynamic knee valgus; and asymmetry indices (LSI, AI) determined on the basis of jump tests. The following tests were accepted: countermovement jump (CMJ), squat jump (SJ), drop vertical jump (DVJ), drop jump (DJ), single-leg vertical jump (SL VJ) and single-leg countermovement jump (SL CMJ), performed in single- and double-leg variants. Studies reporting only subjective clinical scales without objective functional tests were excluded.

With regard to study design, the following were included: randomised controlled trials (RCTs), non-randomised interventional studies, prospective and retrospective cohort studies, analytical cross-sectional studies and case series comprising at least 10 participants. The following were excluded: single case reports, review papers, letters to the editor, conference abstracts without full publication and grey literature not available in a full peer-reviewed version. Only publications in English, published in peer-reviewed journals up to the search cut-off date, were considered.

### 2.3. Selection Process

All results were imported into a shared Excel spreadsheet and RIS files, where deduplication was then performed using EndNote version 2025.3.1 (Clarivate, London, UK). The obtained results were then imported into a shared Excel spreadsheet, which was divided into separate parts to enable individual blinded selection by two co-authors. Two people were selected from among the authors, and the previously established inclusion and exclusion criteria were discussed in order to reach agreement among the authors regarding the intended actions in the selection process. Two authors (K.K. and Ł.R.) performed the selection on the basis of the title, abstract and keywords imported from the databases. Any doubts and conflicts regarding whether or not to accept an article for analysis were resolved by a third author (T.A.) after consultation with the remaining members of the author team.

### 2.4. Data Collection Process

Two authors (Ł.R. and K.K.) independently extracted the data according to a previously adopted standardised scheme, developed and approved by the entire author team. Discrepancies were resolved by a third author (T.A.). The correctness of the extraction was independently verified by a further author (M.N.) on a randomly selected sample of 25% of the included studies.

Data were extracted from the main text, tables and Supplementary Materials. When numerical values were missing, they were read from graphs using WebPlotDigitizer software (v 4.7), or the authors were contacted. Missing data were not imputed and were marked as “n.d.”.

### 2.5. Data Items

Two main data sets were extracted:

- Primary: vertical jump height (cm, in various test variants) and limb asymmetry indices (LSI expressed as %, AI).
- Secondary: peak ground reaction force (vGRF), peak mechanical power (PP), rate of force development (RFDe), concentric- and eccentric-phase impulses, reactive strength (RSI), knee abduction moment (KAM), joint kinematics (flexion and valgus angles) and EMG activity.

Descriptive characteristics were also extracted from each study: authors and year of publication, study design, sample size, age, graft type, follow-up time, jump test used and measurement equipment. The procedure was carried out using a standardised form in a Microsoft Excel spreadsheet. When data were missing, Supplementary Materials were analysed; when unambiguous point values were unavailable, they were obtained from graphs using WebPlotDigitizer software (v 4.7). Missing data were not imputed.

### 2.6. Effect Measures

Because of the large heterogeneity of the included studies, effect measures were selected individually in accordance with the SWiM guidelines:

- Continuous variables on a uniform scale: the mean difference (MD) with a 95% confidence interval (CI) was used.
- Continuous variables on different scales: the standardised mean difference (SMD, Hedges’ g), correcting for small-sample bias, was used. The effect was interpreted according to Cohen: 0.2, small; 0.5, moderate; 0.8, large.
- Limb symmetry index (LSI): point values, measures of dispersion (SD/IQR) and the proportion of patients meeting the return-to-sport criterion (LSI ≥ 90%) were reported. Differences in LSI were analysed using MD.
- Correlations: Pearson coefficients (r) and coefficients of determination (r^2^) were reported. A threshold of *p* ≤ 0.05 was adopted for statistical significance, retaining any author corrections for multiple comparisons.
- Longitudinal studies: effect measures were assessed separately for each time point of 3, 6, 9, 12 and >12 months.

The above measures were planned in case a quantitative synthesis proved feasible. Because the feasibility criteria specified in Section 2.7 were not met, pooled estimates were not calculated. Effect sizes with 95% confidence intervals and *p*-values reported in the source studies were extracted.

### 2.7. Synthesis Methods

A preliminary assessment of the feasibility of a quantitative meta-analysis was performed in accordance with the criteria specified in the Cochrane Handbook [11], evaluating heterogeneity in four categories: clinical (populations, follow-up time, graft type); methodological (study design, measurement protocols, equipment); statistical (dispersion indices and effect measures); and with respect to the reported endpoints. In the second stage, specific feasibility criteria were applied: a subgroup meta-analysis was to be performed where at least three studies jointly met all the following conditions:

(a) the same graft comparison;
(b) the same jump test and its variant;
(c) the same outcome;
(d) a convergent follow-up period;
(e) reporting of means along with a measure of dispersion or data allowing for their reconstruction.

No subgroup met all five criteria, so a meta-analysis was not performed. Because no pooled analysis was conducted, the I^2^ statistic was not calculated; therefore, heterogeneity was described qualitatively.

A structured narrative synthesis in accordance with the SWiM guidelines was adopted as the main method. The studies were grouped in two ways:

1. By graft type;
2. By jump test.

In accordance with the SWiM guidelines, the synthesis was not based on counting the number of studies reaching statistical significance. For each subgroup and time point, the following were reported jointly: sample size, follow-up time, direction of the effect, effect size with its 95% confidence interval, *p*-value and risk of bias assessment for the given study. Studies for which no effect size was reported and for which it could not be calculated post hoc were described qualitatively.

### 2.8. Risk of Bias Assessment

Because of the heterogeneity of the study designs included in this systematic review, several assessment tools were applied in parallel, each tailored to a specific study type, in accordance with the recommendations of the Joanna Briggs Institute (JBI) [12], the Cochrane Collaboration [13] and the Preferred Reporting Items for Systematic Reviews and Meta-Analyses (PRISMA) 2020 guidelines [10]. The quality of all studies and the risk of systematic bias were assessed independently by two co-authors (Ł.R. and K.K.), and any discrepancies were discussed by the whole team and resolved by a third author (T.A.).

#### 2.8.1. Randomised Controlled Trials (RCTs)

These were assessed using the Cochrane Risk of Bias 2.0 (RoB 2) tool [14], which comprises five domains: (D1) bias arising from the randomisation process; (D2) bias due to deviations from the intended interventions; (D3) bias due to missing outcome data; (D4) bias in the measurement of outcomes; and (D5) bias in the selection of the reported result. Each domain was rated as “low risk”, “some concerns” or “high risk”, and the overall assessment was determined in accordance with the guidance proposed by Sterne et al. [14].

#### 2.8.2. Non-Randomised Interventional Studies

The Risk Of Bias In Non-randomised Studies of Interventions (ROBINS-I), version 2 [15], was used; it analyses six domains: confounding, classification of interventions, selection of participants, missing data, measurement of outcomes and selection of the reported result. Each domain and the overall assessment were classified as “low”, “moderate”, “serious”, “critical” or “no information”.

#### 2.8.3. Cohort Studies

The JBI Critical Appraisal Checklist for Cohort Studies [16] was used, while analytical cross-sectional studies were assessed using the JBI Critical Appraisal Checklist for Analytical Cross-Sectional Studies [17]. Case series were assessed using the JBI Critical Appraisal Checklist for Case Series [18]. For each JBI tool, individual criteria were rated as “Yes”, “No”, “Unclear” or “Not applicable”, and the overall methodological quality was classified as low risk of bias (≥70% “Yes” answers), moderate risk (50–69%) or high risk (<50%).

#### 2.8.4. Additional Secondary Assessment for Non-Randomised Studies

All non-randomised studies were independently reassessed using the Methodological Index for Non-Randomized Studies (MINORS) [19], a 12-item list developed specifically for surgical studies. Each item was scored as 0 (not reported), 1 (reported but inadequate) or 2 (reported and adequate); the maximum total score is 16 for non-comparative studies and 24 for comparative studies. MINORS was applied descriptively only, as a supplementary characterisation of reporting in surgical studies, given its widespread use in orthopaedic reviews of ACL reconstruction [19]. MINORS results were not used to derive overall risk of bias judgements, nor to rate the study limitations domain within GRADE. To avoid redundancy between tools, a hierarchy was applied, where for each study the tool appropriate to its design was decisive. In cases where a MINORS score conflicted with the assessment of the primary tool, the primary tool prevailed, and the discrepancy was recorded in the Supplementary Material. Disagreements between the two independent assessors (Ł.R. and K.K.) were resolved through domain-level discussion, and where no consensus was reached, by a third author (T.A.).

### 2.9. Certainty of Evidence Assessment

To assess the certainty of evidence and to guide the strength of the conclusions formulated in this review, the GRADE (Grading of Recommendations, Assessment, Development and Evaluation) approach [20,21] was applied, which corresponds to the recommendation regarding assessment of the certainty of evidence in the guidelines for reporting syntheses without meta-analysis (SWiM). The GRADE approach provides a structured and transparent way of assessing the degree to which the available evidence allows the estimated effect to be considered reliable. Because of the large heterogeneity of study designs, the parallel use of several risk-of-bias assessment tools and the impossibility of performing a quantitative meta-analysis, it was not possible to develop full evidence profiles or summary-of-findings tables based on pooled effect estimates. The GRADE approach was therefore used as a methodological framework for assessing the overall certainty of evidence separately for each key clinical question and the associated vertical jump performance endpoints.

Each of the five downgrading domains was rated as “not serious”, “serious” (one-level downgrade) or “very serious” (two-level downgrade). The starting point was high certainty for clinical questions whose evidence derived predominantly from randomised trials, and low certainty for questions based on observational studies. Certainty could not be downgraded below the very low level (Table 1).

The certainty of evidence was classified into four categories: high, moderate, low and very low [21]. In accordance with the GRADE methodology, high certainty was taken as the starting point for randomised controlled trials and low certainty for observational studies [21,22]. The certainty rating was downgraded when one or more of five conditions occurred: methodological limitations (risk of bias), inconsistency of results, indirectness of evidence, imprecision and publication bias. The possibility of upgrading the rating was considered in accordance with the GRADE guidelines. The results of the risk-of-bias and methodological-quality assessments described in Section 2.8 were used directly to assess the methodological-limitations domain. The certainty of evidence was assessed independently by two authors (Ł.R. and K.K.), and any discrepancies were resolved by a third author (T.A.).

### 2.10. Deviations from the Protocol

The protocol planned a quantitative meta-analysis for comparisons of individual graft types. The meta-analysis was abandoned after data extraction was completed, after finding that no subgroup met the previously defined feasibility criteria; the synthesis was conducted according to the SWiM guidelines. The protocol did not plan for the use of the MINORS tool; it was added post hoc solely as a descriptive characteristic of the quality of reporting in surgical studies, without affecting the risk-of-bias assessment and the GRADE evaluation. None of the above deviations resulted from inspecting the results of individual studies, and none altered the eligibility criteria or the list of endpoints.

## 3. Results

### 3.1. Study Selection

Five electronic databases were searched, identifying 1528 records: Scopus, *n* = 340; MEDLINE, *n* = 425; Web of Science, *n* = 295; PubMed, *n* = 240; SPORTDiscus, *n* = 228. After import into EndNote 2025.3.1 version (Clarivate, London, UK), 582 duplicates were removed, leaving 946 records for screening. Two independent authors (Ł.R. and K.K.) screened titles, abstracts and keywords against the criteria in Section 2.2. At this stage, 289 records were excluded due to the following: no strictly defined graft type (*n* = 175); population < 18 years or ambiguous age (*n* = 73); ineligible publication type, i.e., single case reports, conference abstracts, review papers and letters to the editor (*n* = 41). Full texts were sought for the remaining 657 records. A total of 142 could not be retrieved in English due to lack of institutional access, or full text published only in another language, so 515 reports proceeded to eligibility assessment. Of the 515 full texts assessed, 468 were excluded due to the following: no ACL reconstruction (*n* = 225); no quantitative data allowing extraction (*n* = 132); only subjective clinical scales without objective vertical jump tests (*n* = 90); population after bilateral ACLR or with concomitant reconstruction of other knee ligaments (*n* = 21).

Any discrepancies and doubts between the assessors were resolved by a third author (T.A.) through consultation with the whole team. The inter-rater agreement between the two main reviewers in the full-text phase was 92%, with a Cohen’s kappa coefficient of κ = 0.84. After completing both selection stages, 47 original research studies were included in the final synthesis [2,5,8,23,24,25,26,27,28,29,30,31,32,33,34,35,36,37,38,39,40,41,42,43,44,45,46,47,48,49,50,51,52,53,54,55,56,57,58,59,60,61,62,63,64,65,66].

**Figure 1:**
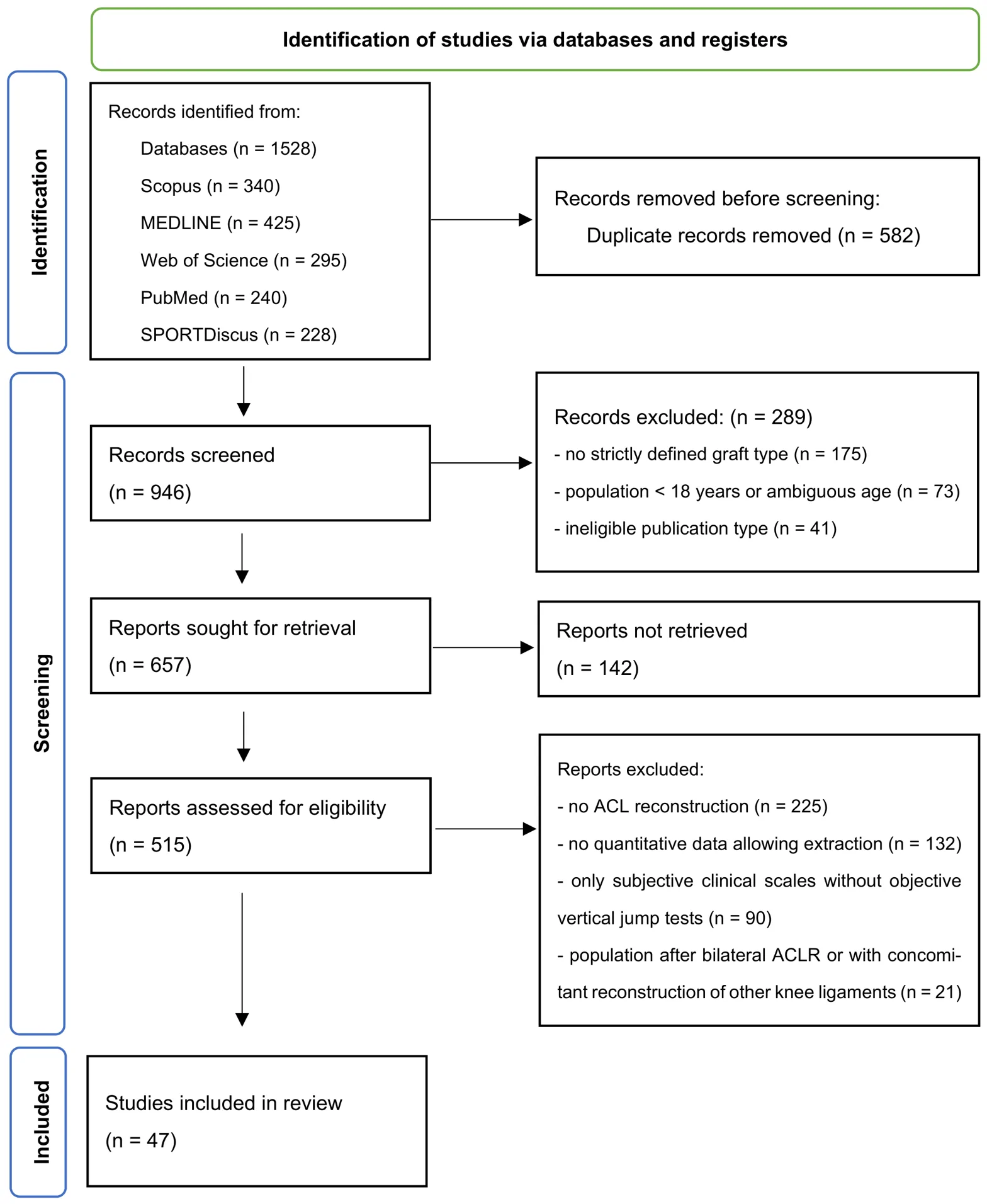
PRISMA flow chart.

The full flow of the review stages, including the number of records identified in each database, the number of duplicates removed, the number of records excluded in the title and abstract phase, the number of full texts assessed for eligibility together with the reasons for exclusion and the number of publications included in the final synthesis, is illustrated in Figure 1.

### 3.2. Study Characteristics

A detailed characterisation of the individual studies included in the review is presented in the Supplementary Table as additional material. Of the 47 included studies [2,5,8,23,24,25,26,27,28,29,30,31,32,33,34,35,36,37,38,39,40,41,42,43,44,45,46,47,48,49,50,51,52,53,54,55,56,57,58,59,60,61,62,63,64,65,66], observational studies predominated; randomised controlled trials constituted a minority, namely 5 papers, corresponding to 10.6% [23,24,25,26,27]. The remainder comprised 21 analytical cross-sectional studies, constituting 44.7% [8,38,46,47,48,49,50,51,52,53,54,55,56,57,58,59,60,61,62,63,64]; 16 cohort studies, 10 prospective and 6 retrospective, constituting 34.0% [2,5,31,32,33,34,35,36,37,39,40,41,42,43,44,45]; 3 interventional studies, constituting 6.4% [28,29,30]; and 2 case series, constituting 4.3% of all publications [65,66].

The included publications spanned the period from 1998 to 2026. As many as 31 of the 47 studies were published after 2018. The studies originated from centres in Europe, North America, Australia and Asia, with the largest number coming from teams in Italy, the United States, Germany, Austria and Australia.

The total number of participants included in the analysis exceeded 3400 people, comprising approximately 2800 patients after ACLR and approximately 600 people in healthy control groups. The size of the individual samples varied, from 12 [30] to 265 [5] participants after ACLR, with a median of approximately 50 patients per study. The largest samples were represented by the studies of Labanca et al. [5] (*n* = 265), Csapo et al. [50] (*n* = 245), O’Malley et al. [58] (*n* = 162), Sun et al. [26] (*n* = 166), Sun et al. [27] (*n* = 156) and Edwards et al. [33] (*n* = 91).

The mean age of participants ranged from 21.3 ± 5.0 [43] to 39.3 ± 32.6 years [55], with a median of approximately 27 years. In accordance with the inclusion criteria, all studies comprised adult patients over 18 years of age. In terms of sex proportions, most studies, as many as 38 of 47, comprised mixed populations of both sexes; 7 papers included only men [8,31,36,40,51,55,58], and 2 studies were limited to women [39,60]. The characteristics comprised both recreationally active patients and competitive athletes, including elite-level alpine skiers [39,40,55], soccer players [29,31] and professional athletes of various disciplines [44].

**Figure 2:**
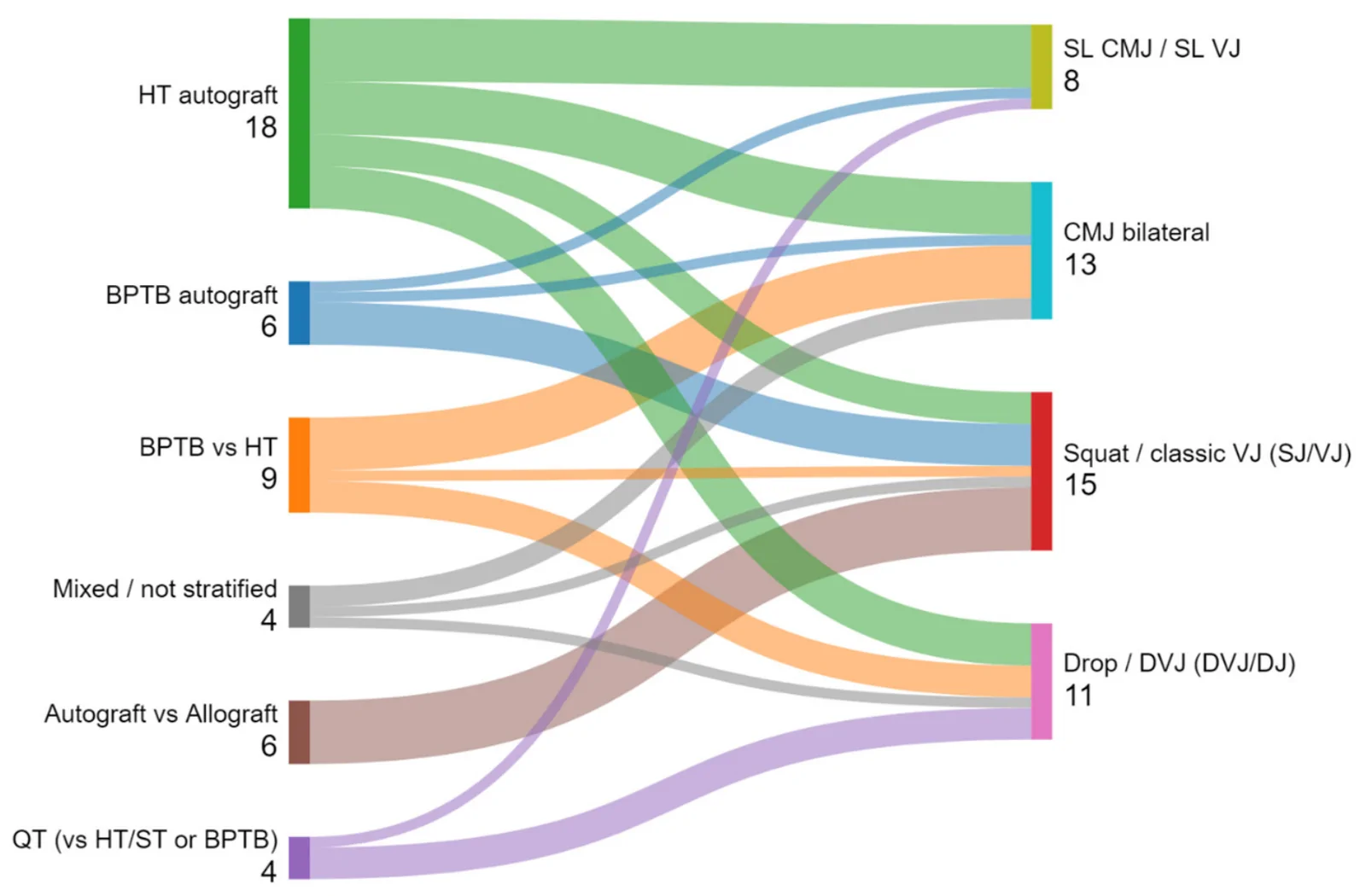
Sankey diagram for group classification.

In terms of graft type, the largest group comprised studies using a hamstring tendon autograft (HT, or its variants ST, STGR, STG); they included 16 papers [2,28,30,31,33,34,35,37,40,41,42,45,49,56,57,63]. Six studies [31,47,51,58,63,65] concerned only the patellar tendon autograft (BPTB). A direct comparison of BPTB with HT/ST was performed in 9 papers [5,8,23,32,40,44,46,61,62], while a comparison of the quadriceps tendon (QT) with HT/ST or BPTB featured in 4 studies [36,50,52,53]. The use of allografts was reported in 5 papers [24,25,26,27,43]; one revision study used a BPTB allograft [66]. In total, the graft categories comprise 47 studies; because of studies reporting more than one graft type, the categories partially overlap, as reflected in the Sankey diagram (Figure 2).

Follow-up times from ACL reconstruction varied and ranged from 3 [44] to 108 months [65]. The studies were classified into four time categories: early postoperative phase (≤6 months), 12 studies; intermediate phase (>6 to 12 months), 13 studies; late phase (>12 to 24 months), 9 studies; and long-term follow-up (>24 months), 13 studies. In total, 8 studies [2,33,35,37,42,45,50,54] were longitudinal and reported results at two or more time points.

The primary tools for assessing vertical jump performance were the countermovement jump in the bilateral variant (13 studies), the single-leg countermovement jump and single-leg vertical jump (7 studies), the drop vertical jump and drop jump (8 studies) and the squat jump (4 studies). Measurements were performed using a variety of equipment, including dual-force plates enabling independent impulse analysis for each limb [5,8,32,33,40,49], three-dimensional motion-capture systems [23,37,46,52,53,60,62,64], optoelectronic systems [34,38,44], accelerometers [61] and the Back-in-Action (BIA) test battery [50,54].

Among the reported parameters, the dominant measures were jump height (cm), reported in 41 of 47 studies; limb symmetry index (LSI, %), in 32 studies; peak ground reaction force (vGRF), in 18 studies; peak mechanical power (PP), in 11 studies; eccentric- and concentric-phase impulses, in 9 studies; and landing kinematic parameters, in 14 studies. Six studies [23,30,40,45,60,64] supplemented the biomechanical measurements with an electromyographic assessment of selected lower-limb muscles.

A detailed list of characteristics of the individual studies, including authors and year of publication, sample size and demographic characteristics, graft type, follow-up time from ACLR, jump test used, measurement equipment, reported outcome parameters, point values together with measures of variability, obtained *p* values and effect sizes, is provided in the Supplementary Material (Supplementary Files S1 and S2). Several included studies originate from the same centres and may involve partially overlapping patient cohorts. This applies in particular to the series [2,8,24,25,26,27,32,38,39,40,52,53,55,58,65,66] which share a centre, a research team and an overlapping recruitment period. The source publications did not provide information allowing the extent of the overlap to be determined. These studies were retained because they reported different outcomes or different follow-up time points, but the potential overlap is recorded in the Supplementary Material and was taken into account when rating certainty of evidence, particularly in the imprecision domain. Aggregate sample sizes reported in this review should therefore be read as the sum of participants across studies rather than as a count of independent patients.

### 3.3. Risk-of-Bias and Quality Assessment Results

In total, 47 studies were included and subjected to methodological quality assessment. The distribution of the assessment tools used was as follows: the RoB 2.0 tool was applied to 5 RCTs [23,24,25,26,27]; ROBINS-I to 3 non-randomised interventional studies [28,29,30]; the JBI checklist for cohort studies to 16 cohort studies [2,5,31,32,33,34,35,36,37,39,40,41,42,43,44,45]; the JBI checklist for analytical cross-sectional studies to 21 studies [8,38,46,47,48,49,50,51,52,53,54,55,56,57,58,59,60,61,62,63,64]; and the JBI checklist for case series to 2 case-series studies [65,66]. MINORS was additionally applied to all 42 non-randomised studies solely to describe reporting completeness; it was not used to derive overall risk-of-bias judgements or to inform GRADE ratings.

**Figure 3:**
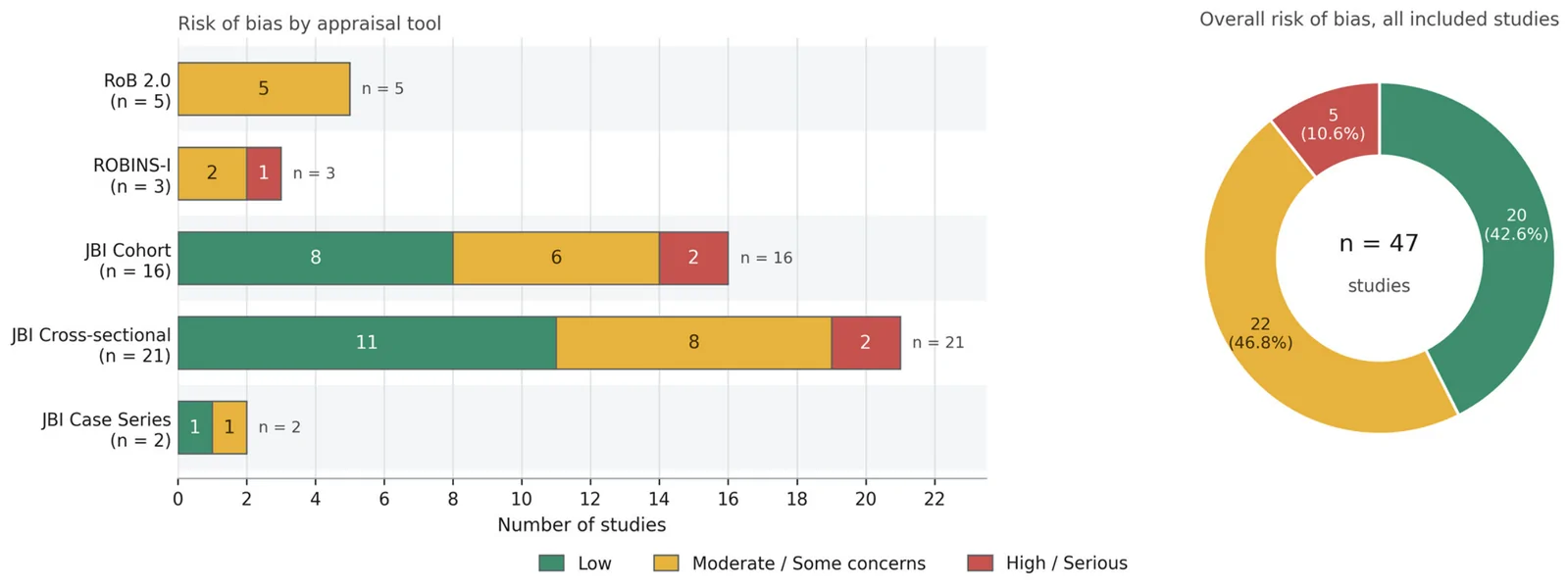
Overall risk of bias.

Throughout the body of evidence (*n* = 47), 20 studies (42.6%) were assessed as having a low overall risk of bias, 22 studies (46.8%) as moderate (or “some concerns” for randomised trials), and 5 studies (10.6%) as high or serious (Figure 3).

The domain least adequately met across the entire set was the control of confounding factors: 21 out of 42 non-randomised studies presented no strategy for managing confounders. Furthermore, in observational studies, the choice of graft was determined by the surgeon based on patient characteristics and athletic demands, introducing a source of confounding by indication.

**Figure 4:**
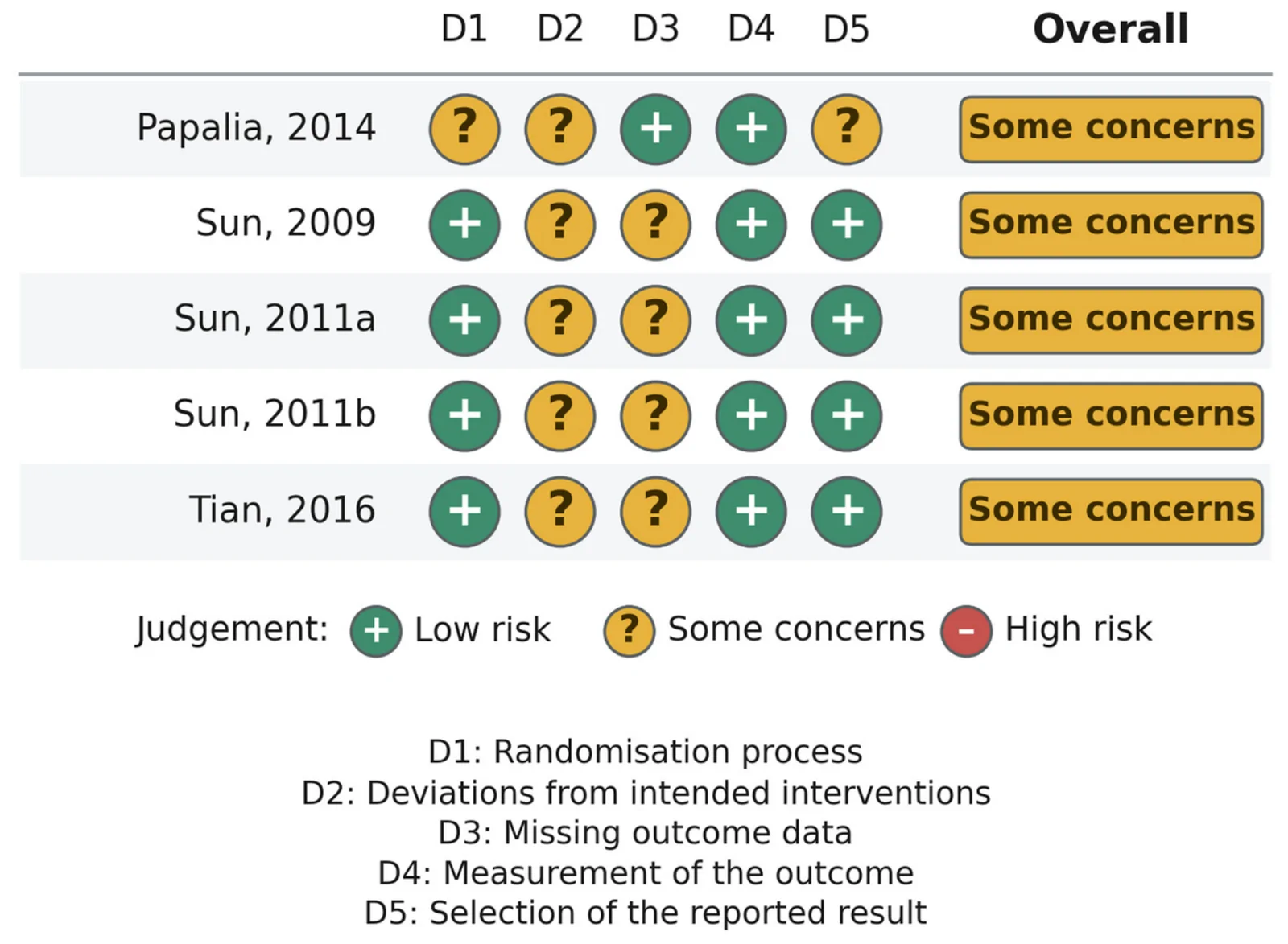
Traffic-light plot of risk of bias for randomised controlled trials [23,24,25,26,27].

All five RCTs [23,24,25,26,27] received an overall rating of “some concerns” (Figure 4). Recurring reservations concerned the lack of a description of allocation concealment (D1) and the impossibility of blinding the surgeon and participants (D2), which is a standard limitation of surgical trials. Domains D3 (missing data), D4 (objective outcome measurement) and D5 (reported results) were generally rated as low-risk. The long-term follow-up periods (3.5–5.6 years) in the studies of Sun et al. [25,26,27] and Tian et al. [24] introduced a potential risk of bias arising from participant attrition, although this was not explicitly discussed.

**Figure 5:**
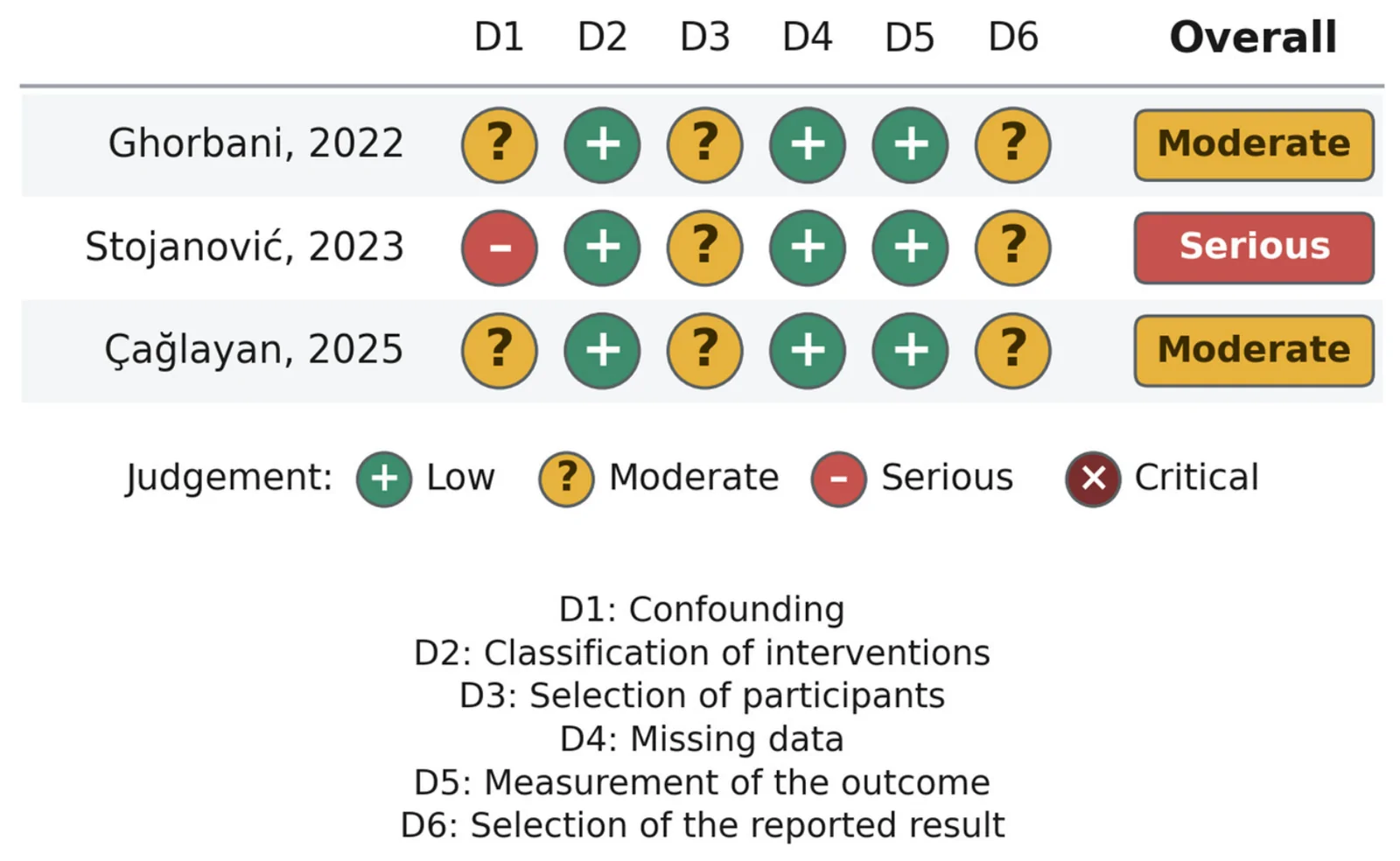
Traffic-light plot of the ROBINS-I V2 scale for non-randomised interventional studies [28,29,30].

Among the three studies assessed using the ROBINS-I tool [28,29,30], two were classified as having a moderate overall risk of bias, while one was classified as having a serious risk of bias (Figure 5). The serious risk of bias in the study by Stojanovic et al. [28] resulted from the confounding domain (D1), as the study combined patients with different graft types without stratification or statistical adjustment. In the other two studies [29,30], the main limitations were the lack of blinding and the absence of a prospectively registered protocol, despite the use of objective, standardised biomechanical measures. Across all three studies, the confounding domain (D1) and the selection-of-participants domain (D3) were the least adequately addressed domains.

**Figure 6:**
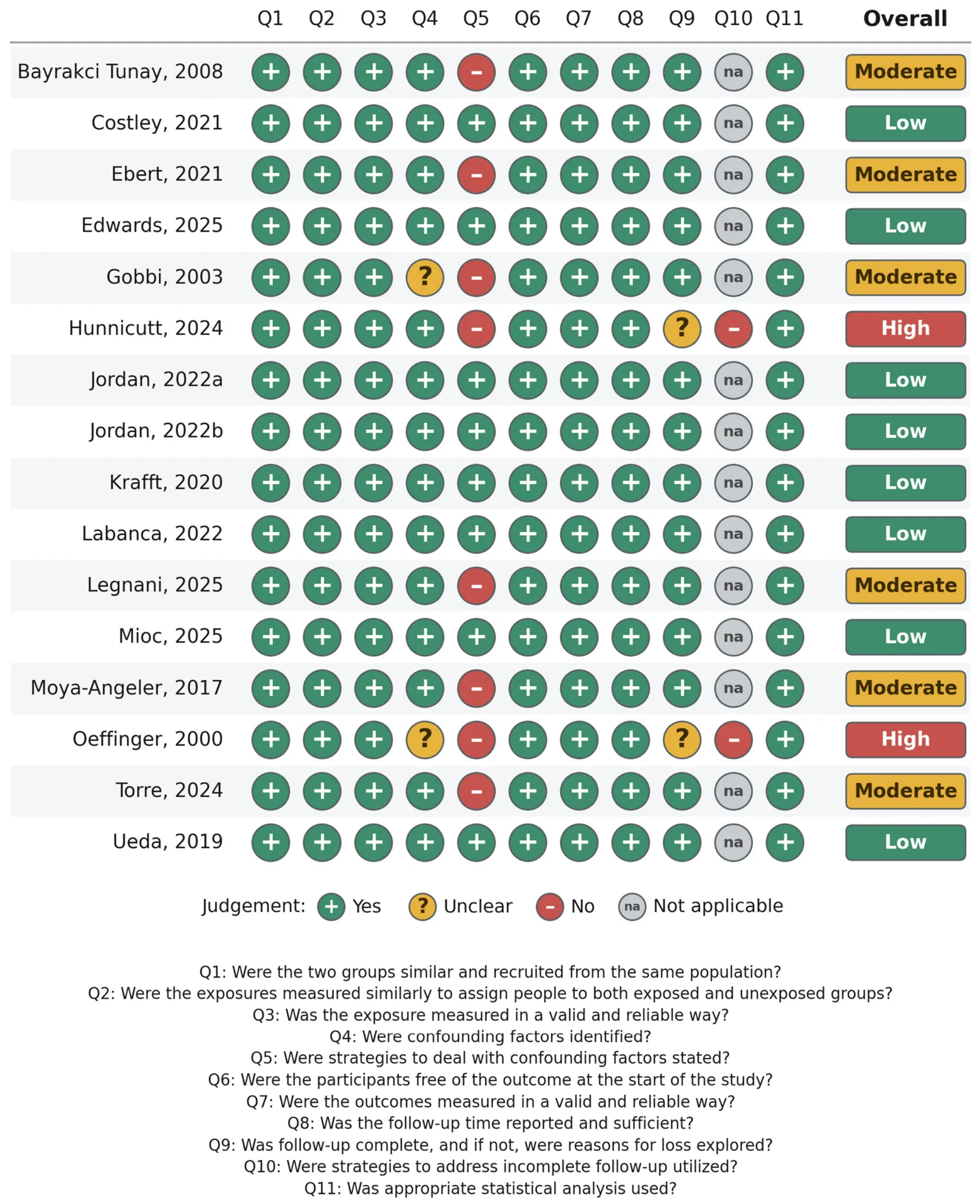
Traffic-light plot of the JBI Critical Appraisal Checklist for Cohort Studies [2,31,32,33,34,35,36,37,39,40,41,42,43,44,45].

Among the 16 cohort studies assessed with the JBI tool (Figure 6), 8 were classified as low risk of bias, 6 as moderate, and 2 as high. The moderate and high ratings were driven predominantly by the confounding domain (Q4–Q5), which was inadequately addressed in 8 of the 16 studies. The most frequently met criteria were reliable outcome measurement (Q7), reliable exposure measurement (Q3) and appropriate statistical analysis (Q11). The most frequent shortcomings concerned Q5 (strategies for dealing with confounding factors) and Q10 (strategies for incomplete follow-up). Larger cohort studies, i.e., Labanca et al. [5] (*n* = 265), Edwards et al. [33] (*n* = 91) and Krafft et al. [37], met all criteria. Item Q10 was recorded as “not applicable” in studies with complete follow-up, because the question about strategies for handling incomplete follow-up does not then apply.

**Figure 7:**
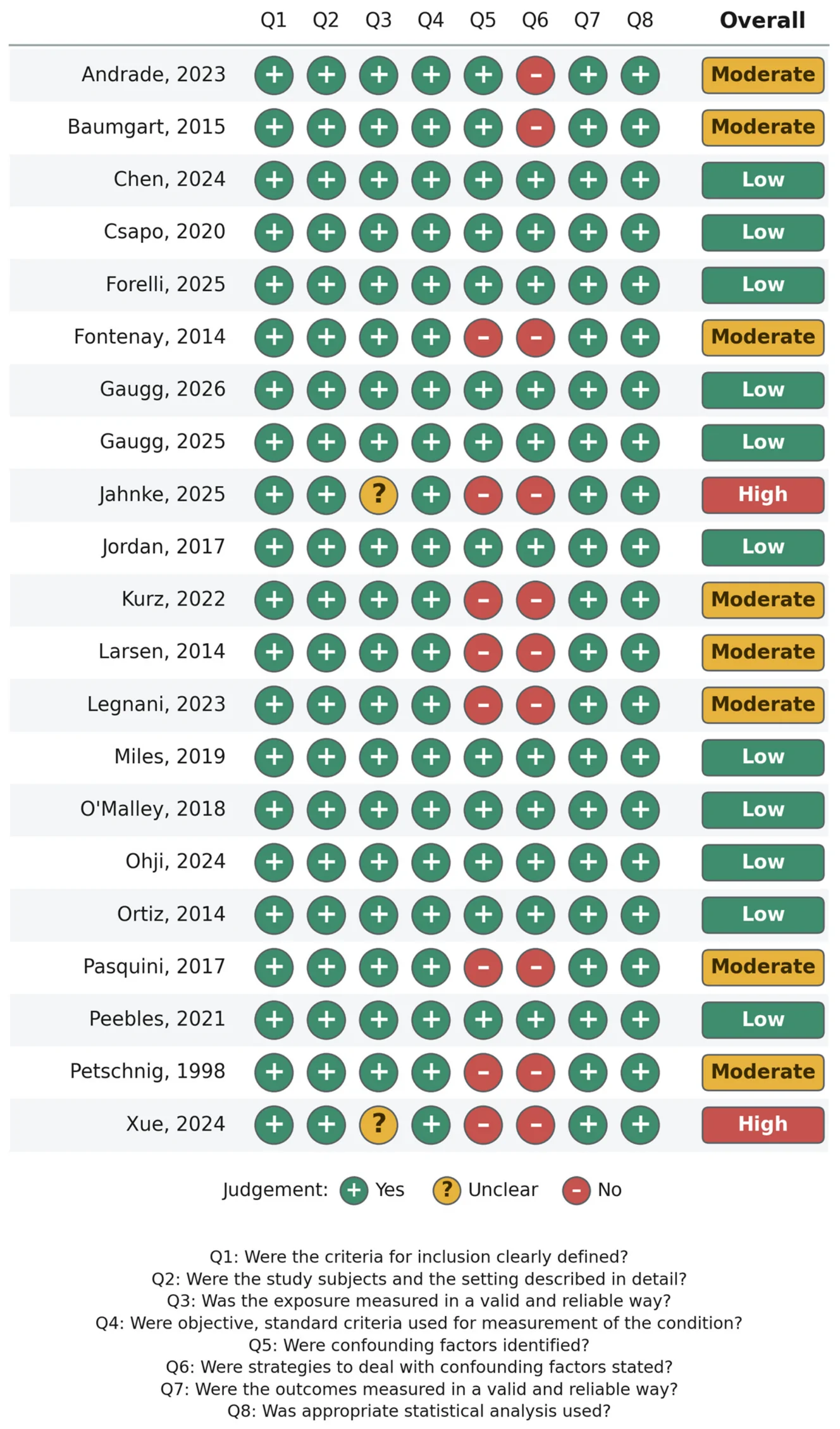
Traffic-light plot of the JBI Critical Appraisal Checklist for Analytical Cross-Sectional Studies [8,38,46,47,48,49,50,51,52,53,54,55,56,57,58,59,60,61,62,63,64].

Of the 21 cross-sectional studies assessed with the JBI tool (Figure 7), 11 were rated low risk of bias, 8 moderate and 2 high. In every study rated moderate or high, the confounding domain (Q5–Q6) was inadequately addressed. Larger studies, including Csapo et al. [50] (*n* = 245) and O’Malley et al. [58] (*n* = 162), achieved the maximum score (8/8). Typical limitations concerned insufficient identification (Q5) and control (Q6) of confounding factors, particularly in pilot or small-sample studies [51,61].

**Figure 8:**
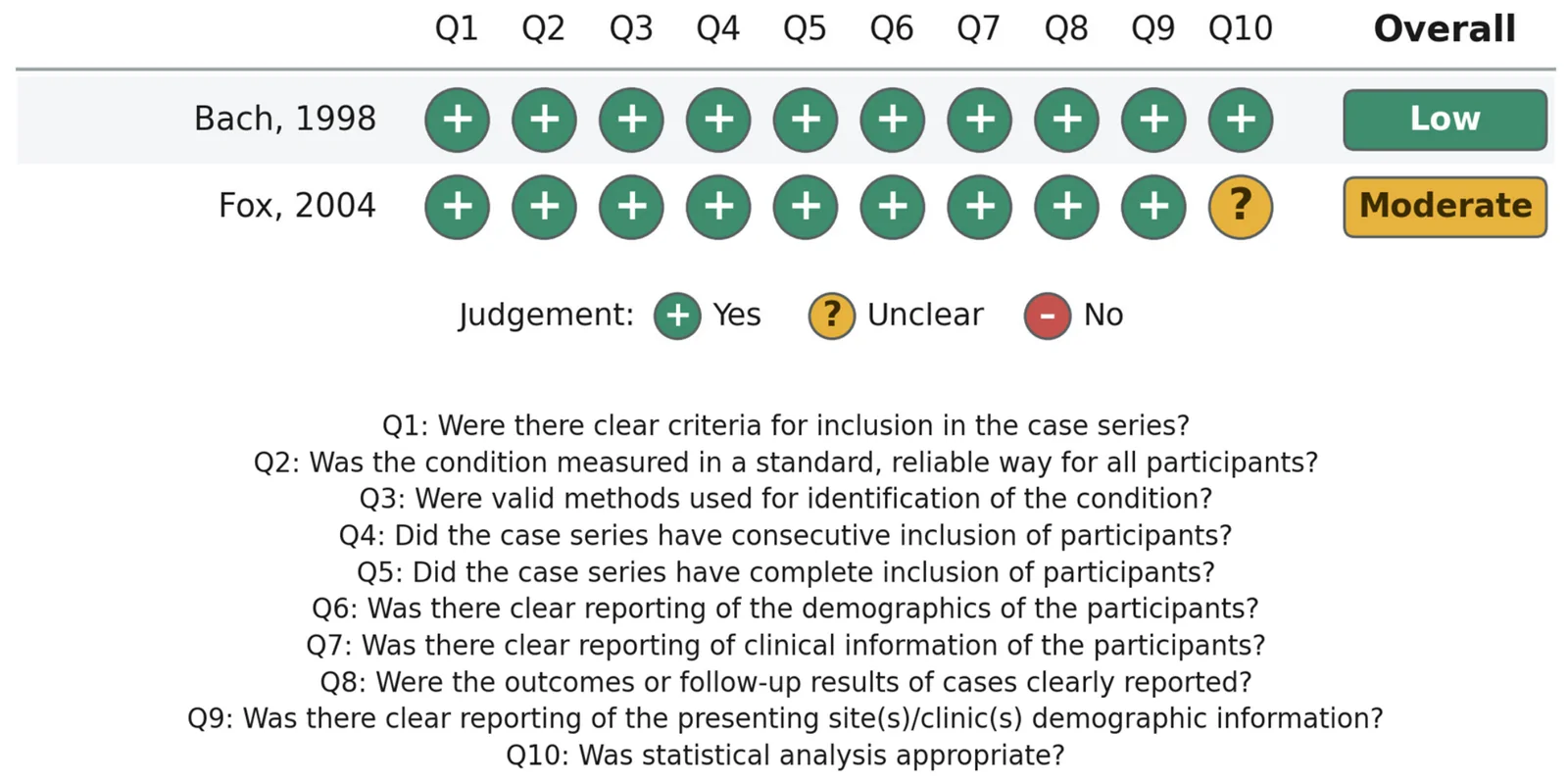
Traffic-light plot of the JBI Critical Appraisal Checklist for Case Series [65,66].

Among the case series (Figure 8), Bach et al. [65] satisfied all ten JBI criteria and was rated at low risk of bias, whereas Fox et al. [66] was rated at moderate risk: the criterion of adequacy of the statistical analysis (Q10) was recorded as unclear because of the very large variability of results reported without further discussion.

**Figure 9:**
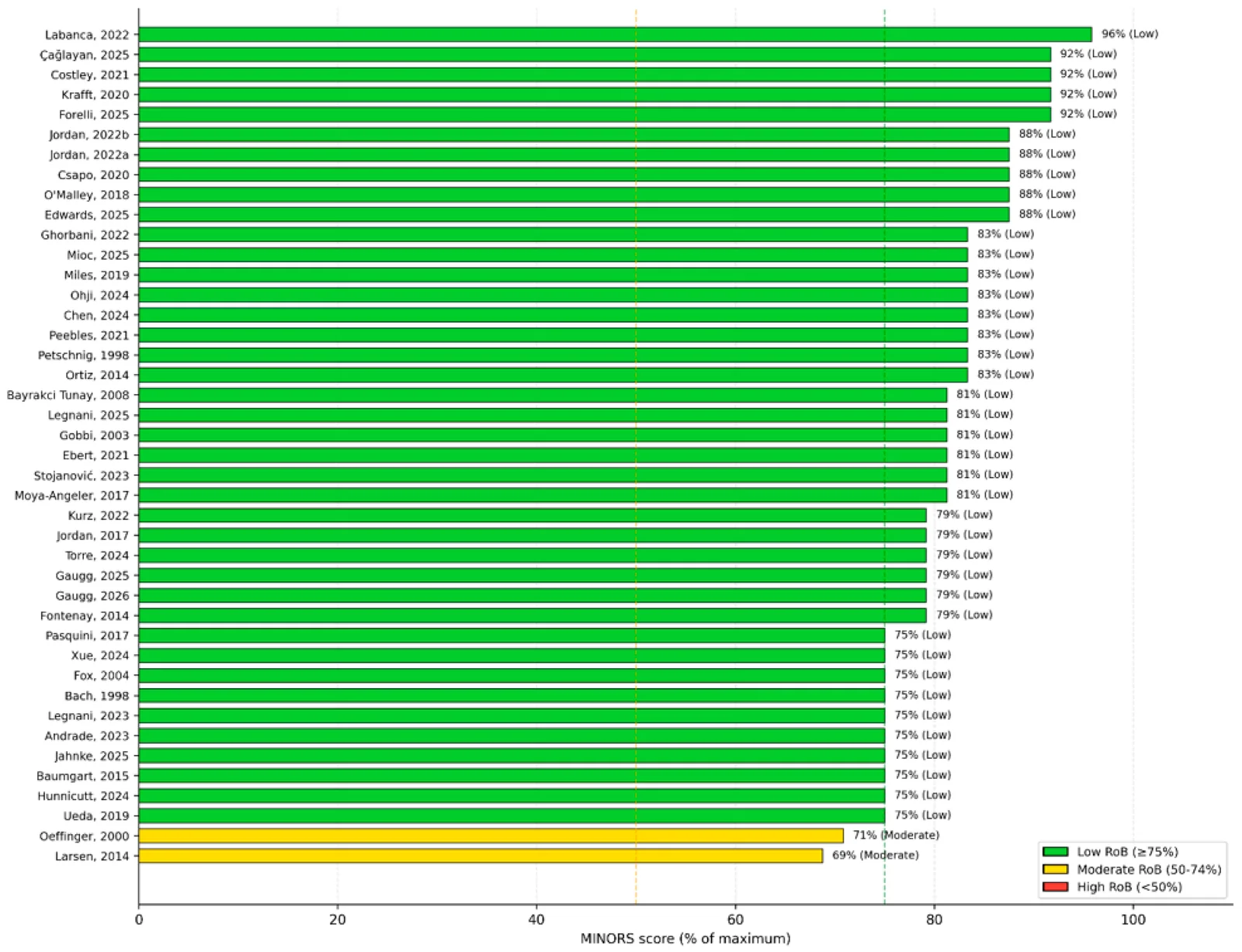
MINORS score distribution [2,5,8,29,30,31,32,33,34,35,36,37,38,39,40,41,42,43,44,45,46,47,48,49,50,51,52,53,54,55,56,57,58,59,60,61,62,63,64,65,66].

Descriptive assessment of all non-randomised studies with MINORS (Figure 9) yielded a median of 17/24 for comparative studies and 13/16 for non-comparative studies. These scores characterise the completeness of reporting and were not used to derive overall risk-of-bias judgements. The highest scores (≥80%) were obtained by the studies of Labanca et al. [5], Edwards et al. [33], Jordan et al. [39] and Costley et al. [32]. The most frequent problems concerned prospective calculation of the sample size (criterion 8) and unbiased assessment of the study endpoints (criterion 5), again reflecting the methodological limitations typical of research on ACL reconstruction [19,67].

### 3.4. BPTB vs. HT Comparison

Of the 47 studies included in the review, 9 were direct comparisons of the BPTB graft with the hamstring tendon graft in the context of vertical jump performance after anterior cruciate ligament reconstruction (ACLR) [5,8,23,32,40,44,61,62]. In total, they comprised more than 600 patients followed for periods of 3 to 24 months after surgery.

In the bilateral countermovement jump, performed on dual-force plates, the BPTB graft was associated with significantly greater eccentric-impulse asymmetry of the operated limb compared with HT at 9 months after surgery (*p* = 0.008) [8]. In the study of Labanca et al. (*n* = 265), at 3 months, patients after BPTB showed higher peak mechanical power but a lower limb symmetry index in both the eccentric and the concentric phase compared with the STGR group (*p* < 0.05); these differences disappeared after 6–9 months [5]. Jordan et al. showed that BPTB causes a significantly higher asymmetry index in both phases of the CMJ than the ST graft throughout the rehabilitation period (*p* < 0.05), with a slower return to control-group values [40].

In the study of Costley et al. (*n* = 44), no significant modulation of concentric- or eccentric-phase CMJ impulse asymmetry by graft type was demonstrated at 6 and 9 months after surgery (*p* > 0.05) [32]. Similarly, in 44 professional athletes studied by Torre et al. at 3 months after surgery, no differences in CMJ LSI between BPTB and HT were shown (LSI = 85.3 ± 8.9%; *p* = 0.56) [44], suggesting that the sporting level may moderate the effect of the graft on performance in the early rehabilitation phase.

In the context of landing biomechanics in the drop vertical jump (DVJ), Papalia et al. (*n* = 40) showed that BPTB combined with a proprioceptive rehabilitation protocol resulted in significantly lower knee abduction moment values than HT at 6 months (*p* < 0.0001) [23], which may argue for a more favourable biomechanical profile. In turn, Peebles et al. observed that BPTB is associated with greater knee-extension-moment asymmetry in the stop-jump test compared with HT (*p* = 0.006) [62]. Andrade et al. did not demonstrate significant kinematic differences in the DVJ between the two graft types [46].

In the single-leg squat jump (SJ), Pasquini et al. showed the opposite direction of difference: at 12 months after ACLR, the height achieved in the single-leg squat jump of the operated limb was significantly higher after BPTB than after HT (*p* = 0.037) [61]. This result suggests that harvesting the hamstring tendons may negatively affect the ability to generate power in the concentric phase.

### 3.5. Autograft vs. Allograft Comparison

Six studies [24,25,26,27,43,66], including four randomised controlled trials [24,25,26,27], comprised a direct comparison of vertical jump performance after ACLR using an autogenous and an allogeneic graft. The total number of patients was 478, and the follow-up period ranged from 2 to 5.6 years.

In the study of Sun et al. (*n* = 156), after 5.6 years of follow-up, no significant differences in vertical jump LSI or in the single-leg hop test were shown between the BPTB autograft and allograft (*p* > 0.05) [27]. Analogous results were obtained for the HT graft in the second study of Sun et al. (*n* = 166), where the autograft and fresh-frozen allograft gave comparable values in VJ LSI [26]. In the study of Tian et al. (*n* = 87), using a double-bundle technique, comparison of the HT autograft with an irradiated allograft showed no significant differences in VJ performance (*p* = 0.677) [24].

In a third study, Sun et al. (*n* = 69) compared irradiated and non-irradiated HT allografts and showed that irradiation worsens knee joint stability but does not affect VJ results (*p* > 0.05) [25]. This observation was confirmed by the results of Tian et al., indicating that knee joint stability and vertical jump performance are independent domains of the ACLR outcome [24].

These results complement the observations of Oeffinger et al. (*n* = 23), where, at 21–28 months, the heights achieved in the single-leg VJ did not differ significantly between the BPTB autograft (38.5 ± 5 cm) and allograft (36.8 ± 6 cm), and both groups fell within the range of results achieved in the control group (39.9 ± 6 cm; *p* = 0.47) [43]. In revision ACL reconstruction using a BPTB allograft, Fox et al. (*n* = 32) reported a mean VJ LSI of 98%, but with very high variability of results (SD = 33.2%; range 35–232%) [66].

The available evidence, on a combined sample of 478 patients, indicates that the choice between autograft and allograft, regardless of preparation, whether fresh-frozen or irradiated, does not affect vertical jump performance after ACLR. The clinical decision should therefore be based on criteria such as knee joint stability, the risk of reoperation and patient preferences, rather than on potential jump performance achievable in the future.

### 3.6. Countermovement Jump

The bilateral countermovement jump, performed most often on dual-force plates enabling independent impulse analysis of each limb, was the most frequently used assessment tool in the included studies (13 papers) [5,8,28,32,33,37,39,40,41,47,48,49,57].

Patients after an HT autograft showed, at 6 months after surgery, a reduced LSI for ground reaction force of 85.9 ± 9.6% compared with 94.9 ± 5.3% in healthy individuals (*p* < 0.001), with the largest deficits in the braking phase (68.0 ± 23.1% vs. 76.7 ± 17.2%) [49]. Edwards et al., in a sample of 91 patients after an HT graft, observed that between 6 and 9–12 months after surgery, CMJ height increased by 1.46 cm, and the concentric- and eccentric-phase asymmetry indices decreased by 3.13% and 3.59%, respectively (*p* ≤ 0.05) [33]; however, the reduction in asymmetry resulted mainly from a decrease in values on the non-operated side rather than from improvement on the operated side [33].

Krafft et al., assessing patients from the preoperative state to 6 months after HT, showed that maximum knee-extension moments in the CMJ increase in stages but do not reach the values observed in the healthy group (*p* < 0.05) [37].

The characteristics of CMJ deficits depended on the graft type. Miles et al. (*n* = 66) showed that BPTB causes significantly greater eccentric-phase impulse asymmetry than HT at 9 months (*p* = 0.008) [8], and that quadriceps strength is a predictor of concentric-phase asymmetry (BPTB: r^2^ = 0.39; HT: r^2^ = 0.18) [8]. Labanca et al. (*n* = 265) confirmed that BPTB is associated with higher peak power but greater asymmetries at 3 months compared with STGR; the differences equalised after 6–9 months [5]. In turn, Costley et al. did not find a significant change in asymmetry due to the graft (*p* > 0.05) at 6 and 9 months [32].

In long-term follow-up, Jordan et al. showed that asymmetry indices normalise approximately 2 years after ACLR, but jump height and peak power values remain reduced for up to 5 years (*p* < 0.01) [39]. Baumgart et al., in patients approximately 31 months after BPTB, identified three compensatory strategies with respect to ground reaction force, the intensity of which correlated with the subjective knee assessment on the IKDC scale [47].

Particularly importantly, Chen et al. (*n* = 70) showed that even in patients meeting the LSI ≥ 90% threshold in the single-leg vertical jump test, CMJ biomechanics in the bilateral variant remains abnormal at approximately 16 months after surgery (*p* < 0.001) [48], indicating that meeting the LSI criterion in single-leg tests does not guarantee full normalisation of vertical jump function [48]. Stojanović et al. additionally showed that eccentric training in late rehabilitation significantly increases CMJ height regardless of graft type [28].

### 3.7. Single-Leg Countermovement Jump/Single-Leg Vertical Jump

The SL CMJ and SL VJ tests were analysed in six studies [2,34,50,58,59,65] comprising a total of more than 500 patients. They were characterised by the greatest sensitivity in detecting asymmetry among all the functional tests used after ACLR.

In the study of Ebert et al. (*n* = 50, HT autograft), at 10 months after surgery, the LSI for the SL CMJ was 83.4 ± 9.5% [34]. Only 18% of patients reached the clinically satisfactory LSI ≥ 90% threshold [34]. O’Malley et al., in a larger sample (*n* = 162), showed an effect size for differences in SL CMJ height of d > 0.4 and an even larger one for LSI (d > 1.1; *p* < 0.001) between patients and controls [58]. In the study of Csapo et al. (*n* = 245, QT vs. HT), at 6 months, 24.5% of patients had difficulty performing the single-leg SL SJ for the operated limb, and 47.3% had at least one test classified as poor or very poor; no significant differences were shown between QT and HT (*p* > 0.05) [50].

In the study of Legnani et al., after an HT graft, a significant increase in SL CMJ height was observed between 6 and 12 months (*p* < 0.01); however, asymmetries persisted at 6 months, and the LSI did not correlate at any time point with RTS at the pre-injury level [2]. The data of Bach et al. (*n* = 97, BPTB autograft) indicate that at 5–9-year follow-up, the asymmetry in the single-leg VJ is <2% (LSI ≈ 98%), suggesting full functional recovery in long-term follow-up, regardless of early deficits [65].

### 3.8. Drop Jump/Drop Vertical Jump

The drop vertical jump (DVJ) and drop jump (DJ) tests were used in eight studies [23,36,42,46,53,60,62,64] to assess landing biomechanics and neuromuscular control of the knee after ACLR. Because of the high eccentric loads, this test is particularly important for identifying patients at increased risk of re-injury of the ACL.

With regard to comparisons of different graft types, Papalia et al., in an RCT (*n* = 40), showed that BPTB combined with a proprioceptive rehabilitation protocol results in lower knee abduction moment values during DVJ landing compared with HT at 6 months (*p* < 0.0001), suggesting a more favourable biomechanical profile in the context of dynamic valgus risk [23]. In turn, Peebles et al. (*n* = 38) observed that BPTB is associated with greater knee-extension-moment asymmetry compared with HT at approximately 6 months (*p* = 0.006) [62].

Andrade et al., in a small sample (*n* = 25), did not demonstrate significant differences in knee kinematics during the DVJ between HT and BPTB (valgus angle *p* = 0.187; rotation *p* = 0.601; flexion *p* = 0.393) [46]. However, patients after ACLR showed reduced ground reaction force compared with healthy individuals (*p* = 0.049; d = 0.84), regardless of graft type [46].

Gaugg et al. showed that at approximately 8 months after surgery, patients after a QT graft achieved smaller dynamic knee valgus during the DVJ (angle +3.5°, IQR −9.1 to 6.3) compared with the ST graft group (angle −2.4°, IQR −11.3 to 3.4; *p* = 0.027) [52]. The mechanism of this difference may be related to preservation of the medial part of the hamstring tendons when QT is harvested, which may provide greater knee joint stability in the frontal plane. In turn, Hunnicutt et al. (*n* = 24, QT vs. PT) did not demonstrate significant biomechanical DVJ differences between QT and BPTB; both groups unloaded the operated limb in an identical way (peak vGRF 1.10 N/kg body mass; *p* > 0.05) [36]. In another study, Gaugg et al. (*n* = 26) did not find ST vs. QT differences in the LSI for the tests but noted that women showed greater contralateral valgus regardless of graft (*p* = 0.016) [53]. In women after HT, Ortiz et al. observed a tendency towards greater dynamic valgus in the DJ at 1–5-year follow-up, although the difference did not reach statistical significance (*p* = 0.07) [60].

### 3.9. Squat Jump

The squat jump (SJ) test, comprising only the concentric phase of the take-off without a countermovement, was used in four studies [51,55,56,61] as a tool for assessing the ability of the knee extensors to generate power after ACLR.

Clinically important data on the comparison of grafts in the SJ test come from the study of Pasquini et al. (*n* = 30, BPTB vs. HT) [61]. At 12 months after surgery, the SJ height of the operated limb was significantly higher after BPTB than after HT (*p* = 0.037) [61], which is the opposite direction of difference relative to the bilateral CMJ, where BPTB causes greater asymmetries. Fontenay et al. (*n* = 13 ACLR after BPTB + 16 healthy), in an analysis of the single-leg SJ, showed deficits at 6–9 months: the LSI of the flight-phase height (Hflight) was 65% vs. 89% in healthy individuals (*p* < 0.001), and the LSI of the total mechanical joint work (WTotal) was 76% vs. 99% (*p* = 0.02) [51]. Interestingly, the deficits arose mainly from the hip and ankle rather than the knee, which may indicate compensatory unloading of the operated knee joint by adjacent segments of the lower limb. Kurz et al. [56] (*n* = 17 ACLR + 67 healthy) showed that at approximately 11 months after HT, despite clinical return to sport, the peak power of the single-leg SJ and the rebound efficiency of the DJ remain lower than in healthy individuals (*p* < 0.05) [56].

### 3.10. Directional Synthesis for Individual Aspects

Because of the high methodological heterogeneity, a formal meta-analysis covering all studies was not undertaken. Nevertheless, for three key clinical questions, the data were sufficiently consistent to allow a targeted narrative synthesis. The synthesis below should be read as a structured description of the direction and magnitude of effects. Because several studies reported neither effect sizes nor confidence intervals, the evidential weight of individual observations is unequal. For this reason, the number of studies and the risk-of-bias rating are stated alongside each conclusion.

#### 3.10.1. CMJ Asymmetry in the BPTB vs. HT Comparison

A synthesis of evidence from seven studies comparing BPTB and HT in the CMJ test [5,8,32,40,44,61,62], comprising a total of >450 patients, was performed.

In the early rehabilitation phase of approximately 3 months, BPTB was associated with higher eccentric-phase asymmetry than STG (*p* < 0.05) [5], and at 9 months with higher eccentric-impulse asymmetry (*p* = 0.008; *n* = 66) [8]. Neither study reported a confidence interval for the between-graft difference. Jordan et al. reported higher CMJ asymmetry indices after BPTB across the rehabilitation period (*p* < 0.05), with a slower return to control-group values [40]. Some studies did not show modulation by the graft, which may result from smaller samples or a specific population type [32,44].

**Figure 10:**
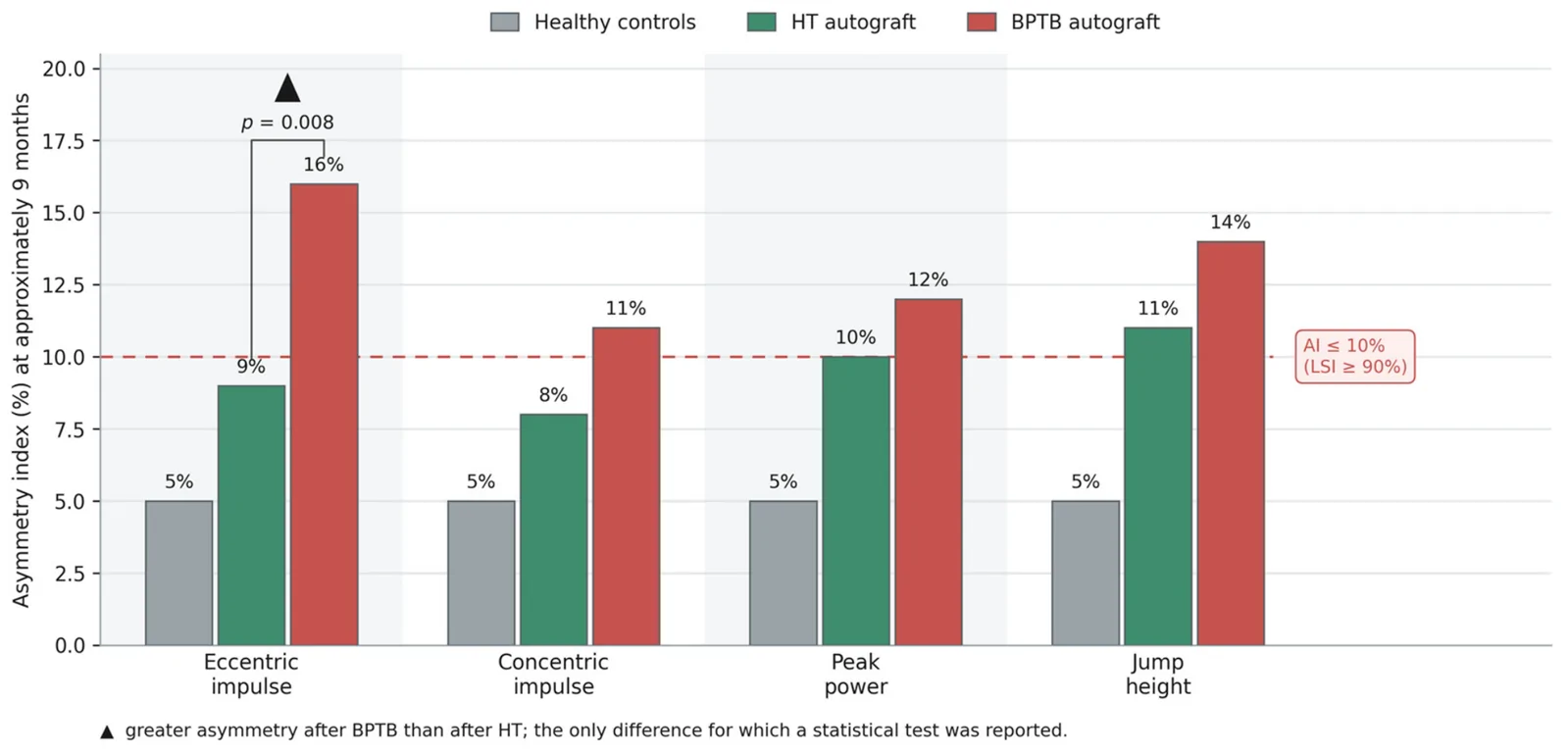
Phase-specific CMJ asymmetry—BPTB vs. HT.

The greater eccentric-phase asymmetry after BPTB probably results from temporary impairment of the knee-extensor apparatus after patellar tendon harvesting, which affects the braking phase of the jump. Two of the eight studies found no association between graft type and CMJ asymmetry [32,44], so the direction of effect is not consistent across the available evidence base. The proposed explanation below is an interpretation by the present authors: none of the included studies tested the underlying mechanism directly. Because certainty of evidence for this question is very low, the observation does not by itself justify graft-specific modification of rehabilitation protocols (Figure 10).

#### 3.10.2. Jump Performance in the QT vs. HT Comparison

Three studies directly compared QT with HT or its variant ST: Csapo et al. [50] (*n* = 245), Gaugg et al. [52] (*n* = 26) and Gaugg et al. [53] (*n* = 26). In total, they comprised approximately 300 patients.

In the SL CMJ test at 6 months after surgery, no significant differences in vertical jump performance between QT and HT were shown according to Csapo et al. (*p* > 0.05) [50]. Demographic factors (age > 19 years, male sex) proved to be more important determinants of the outcome than graft type. Likewise, no ST vs. QT differences were found in the hop test [53].

An important difference, however, was landing biomechanics in the DVJ test. Gaugg et al. showed that patients after a QT graft presented smaller dynamic valgus of the operated knee (angle +3.5°, IQR −9.1 to 6.3) compared with the ST group (angle −2.4°, IQR −11.3 to 3.4; *p* = 0.027) [53]. This result is clinically important because dynamic valgus is a risk factor for re-injury of the ACL.

The available data do not indicate differences in vertical jump height between QT and HT. The observation of smaller dynamic valgus after QT derives from a single study involving 26 patients, with wide interquartile ranges and without adjustment for confounders, including sex, which the same research group identified as a significant predictor of contralateral valgus [53]. Certainty of evidence for this comparison is low. This observation should therefore not be regarded as an argument for selecting a QT graft and requires independent confirmation in adequately powered studies.

#### 3.10.3. Temporal Course of Jump Deficits

The temporal picture presented below is derived predominantly from a juxtaposition of findings across different studies, involving different rehabilitation protocols and measurement methods. Of the 47 included studies, only 8 [2,33,35,37,42,45,50,54] were longitudinal and reported outcomes at two or more time points, and none spanned the full range from the early phase to multi-year follow-up. This description should not be interpreted as a recovery trajectory for an individual patient. Differences between successive phases may partly reflect differences between the populations studied. Vertical jump performance deficits after ACLR have been reported across a range of follow-up intervals. In the early postoperative phase (3 months), all patients, regardless of graft, show limb asymmetries, with a CMJ LSI of around 85% [44]. Differences between graft types are then most pronounced, with BPTB causing greater asymmetries than HT [5].

In the intermediate phase (6 months), deficits persist: the LSI vGRF for the CMJ is approximately 86% [49], and the LSI for the SL CMJ is 83% [34,50]. Only approximately 18% of patients reach the LSI ≥ 90% threshold [34]. Biomechanical asymmetries in the DVJ still persist [42,53]. Differences between BPTB and HT begin to disappear in some studies [5,32].

In the late phase (9–12 months), CMJ jump height improves moderately (+1.46 cm) [33], with the dominant mechanism of asymmetry reduction resulting from a decrease in the performance of the healthy limb. SL CMJ performance improves significantly [2]. The LSI for the SL VJ becomes a good indicator of achieving RTS at the pre-injury level (*p* < 0.05) [59].

In the phase beyond 12 months, asymmetry indices normalise approximately 2 years after surgery [39]; however, stretch-shortening-cycle deficits for jump height and peak output mechanical power may persist for up to 5 years (*p* < 0.01). GRF asymmetries persist in patients with a low IKDC score at approximately 31 months after BPTB [47]. Full normalisation of the LSI for the VJ (>98%) is achieved only at 5–9-year follow-up [65].

This summary is largely cross-sectional, as the data suggest that limb symmetry indices and absolute values may normalise at different rates, which supports considering both types of measurements. The available evidence does not, however, identify a time point or set of values at which return to sport is safe; none of the included studies validated vertical jump measures against re-injury risk.

### 3.11. Certainty of Evidence

Certainty of evidence was rated separately for six clinical questions; the ratings and the justification for each downgrade are presented in Table 1. The starting point was low certainty for five questions, because 42 of the 47 included studies were non-randomised. Only for the autograft-vs.-allograft comparison did the evidence derive predominantly from randomised trials, and the starting point was high certainty. The certainty rating was downgraded for three reasons. Inconsistency of results was a significant limitation, since for most questions, divergent directions and magnitudes of effects were observed, and the high methodological heterogeneity precluded a quantitative synthesis. Indirectness of evidence resulted from the diversity of populations, different graft types and surgical techniques, as well as the heterogeneity of the jump tests used and the reported parameters; moreover, vertical jump performance is only an indirect indicator of sports function and readiness to return to sport. Imprecision concerned studies with small sample sizes and wide ranges of results variability. Upgrading conditions did not apply to any of the analysed endpoints.

The comparison of autograft with allograft was characterised by the greatest consistency and certainty of evidence in this review. Six studies [24,25,26,27,43,66], including four randomised controlled trials [24,25,26,27] comprising a total of 478 patients, consistently showed no significant differences in vertical jump performance regardless of graft type and allograft preparation method. The certainty for this question was downgraded from high to low because of “some concerns” in the risk-of-bias assessment and indirectness of evidence.

For the BPTB-vs.-HT comparison in the countermovement jump, comprising seven studies [5,8,32,40,44,61,62] (>450 patients), a consistent direction of effect was observed, indicating greater eccentric-phase asymmetry after BPTB in the early and intermediate rehabilitation phase; however, because of the predominance of observational studies and the partially divergent results [32,44], the certainty of evidence was rated as low to very low. Similarly, the temporal course of deficits, despite a consistent direction, was based mainly on observational and longitudinal studies, corresponding to low/very low certainty. The lowest certainty was assigned to the QT-vs.-HT comparison, based on a small number of direct studies [52,53,54] with heterogeneous results, and to the observation of a lack of differentiation of jump height itself by graft type.

Because certainty of evidence did not exceed the low level for any outcome examined, the strength of the conclusions and clinical implications arising from this review is conditional. The associations described should be regarded as preliminary and require confirmation in prospective randomised trials with standardised assessment protocols and time points (Table 1).

**Table 1:** GRADE assessment of the certainty of evidence.

Outcome/Comparison	*n* of Studies	Risk of Bias	Inconsistency	Indirectness	Imprecision	Publication Bias	Summary of Findings	Certainty
Vertical jump performance (jump height, LSI): autograft vs. allograft	6 [24,25,26,27,43,66]	Serious	Not serious	Serious	Not serious	Undetected	No difference in vertical jump performance or limb symmetry between autograft and allograft at mid- and long-term follow-up; direction consistent across all six studies.	⨁⨁◯◯ LOW
Eccentric-phase asymmetry in the bilateral CMJ: BPTB vs. HT	8 [5,8,23,32,40,44,61,62]	Serious	Serious	Serious	Serious	Undetected	Greater eccentric-phase asymmetry after BPTB in the early and intermediate phase in most studies, but two [32,44] found no difference; no pooled estimate.	⨁◯◯◯ VERY LOW
Vertical jump height: BPTB vs. HT	8 [5,8,23,32,40,44,61,62]	Serious	Serious	Serious	Serious	Undetected	No consistent difference in jump height between grafts; direction varied across studies and test variants.	⨁◯◯◯ VERY LOW
Vertical jump height: QT vs. HT/ST	3 [50,52,53]	Serious	Not serious	Serious	Serious	Undetected	No difference in jump height between QT and HT/ST at 6 months; demographic factors were stronger determinants of outcome than graft type.	⨁◯◯◯ VERY LOW
Dynamic knee valgus during the DVJ: QT vs. ST	1 [52]	Serious	Not assessable	Serious	Very serious	Undetected	A single small study reported smaller dynamic valgus of the operated knee after QT than after ST; unadjusted for sex, which the same group identified as a predictor of valgus.	⨁◯◯◯ VERY LOW
Temporal course of vertical jump deficits after ACLR	8 [2,33,35,37,42,45,50,54]	Serious	Serious	Very serious	Serious	Undetected	Deficits persist for many months and, in some observations, for several years; symmetry indices and absolute values appear to normalise at different rates. No consistent recovery trajectory could be defined.	⨁◯◯◯ VERY LOW

## 4. Discussion

This systematic review synthesises the available evidence on the effect of the type of graft used in anterior cruciate ligament reconstruction on vertical jump performance in adult patients after ACLR. On the basis of 47 studies comprising more than 3400 participants, of which none provided high-certainty evidence, any effect of graft type appears to be conditional on the time from surgery, the type of functional test and the biomechanical parameter analysed. Graft type does not unequivocally differentiate jump height itself under all testing conditions, but it may influence the profile of deficits revealed in dynamic tasks [32,33,34,37,47,48,49,58]. The most consistent differences concerned comparisons of BPTB and HT in the countermovement jump, especially with respect to eccentric-phase asymmetry in the early and intermediate rehabilitation period [5,8,40]. The results concerning QT and comparisons of autografts with allografts were less unequivocal and should be interpreted in the context of the limited number of studies [24,25,26,27,43,50,52,53,66]. The relationships assessed in this work and their practical implications are discussed in detail below.

Evidence-based findings, i.e., results directly reported in the included studies, are stated in the indicative mood with references to the specific papers and with the corresponding certainty rating. Certainty of evidence was rated low or very low for every clinical question; with a body of evidence of this kind, none of the relationships discussed can be regarded as established or causal.

### 4.1. Graft-Related Functional Adaptations

The type of graft used appears to modify the postoperative profile of functional adaptations; however, the available data do not indicate that any graft unequivocally determines vertical jump performance. The most consistent pattern concerned the comparison of BPTB with hamstring tendon grafts, in which the differences emerged primarily in tasks requiring dynamic load absorption. In studies using the bilateral CMJ, patients after BPTB more often presented greater eccentric-phase impulse asymmetry and loading of the operated limb [5,8,40]. From a biomechanical perspective, this pattern may reflect a temporary limitation of the knee-extensor apparatus after graft harvesting, particularly in the braking phase, in which the limb must rapidly accept load [68]. Alternative explanations remain equally plausible, including differences in rehabilitation protocols or in baseline quadriceps strength.

This does not, however, imply superiority of HT over BPTB with respect to jump function. The lack of significant differences in some CMJ analyses [32,44] and the opposite direction of results in the single-leg squat jump [61] indicate that the “effect” of the graft depends on the nature of the task. The CMJ reveals to a greater extent deficits related to eccentric control and asymmetric limb loading during dynamic movement, whereas the SJ better reflects the ability for concentric force generation [69]. Differences between grafts may therefore not result from higher or lower limb performance but from a different neuromechanical strategy used depending on the test demands [69,70].

Data on QT are less numerous but suggest a potentially different adaptation profile. The results of Csapo et al. did not indicate significant differences between QT and HT in single-leg tests at 6 months, whereas Gaugg et al. showed smaller dynamic knee valgus after QT compared with ST during the DVJ [50,52,53]. Such a pattern suggests that QT may not directly improve jump height but may be associated with a different organisation of movement control in the frontal plane during landing. Because of the limited number of studies, this interpretation should be treated with caution; however, from a biomechanical perspective, it remains important, because the assessment of sports function after ACLR should not be based solely on jump height but also on performance, symmetry and movement-control parameters [69].

Comparisons of autografts and allografts did not show clear differences in vertical jump performance in medium- and long-term observations [24,25,26,27,43,66]. Importantly, in some studies, differences in knee joint stability did not translate directly into the vertical jump result [24,25]. This indicates that mechanical stability and the ability to perform a dynamic task represent partly separate domains of the outcome after ACLR [71]. From a functional perspective, graft choice should not be interpreted as an independent predictor of vertical jump performance. It is more likely that it influences the way in which the ability to generate force, absorb load and control movement during dynamic and sport-specific tasks is recovered [72].

### 4.2. Recovery over Time After ACLR

Recovery of vertical jump performance after ACLR does not proceed linearly. The results of the included studies indicate that individual components of the jump may normalise at different rates. In the early postoperative phase, functional deficits were observed in various patient groups regardless of graft type, with the differences between grafts being most visible in the first months of rehabilitation, especially with respect to asymmetry and control of operated-limb loading [5,8,44]. This may result from limited load tolerance or from protective movement strategies that affect the performance of dynamic tasks even when the patient is formally able to perform the jump test [67].

In the intermediate phase, comprising approximately 6–12 months after ACLR, jump-function deficits still remain clinically important. Studies indicate persisting limitations in ground reaction force, control of the braking phase and single-leg test results, especially in patients after HT or QT or in populations assessed for return to activity [34,49,50]. This means that the mere passage of time from surgery should not be treated as a sufficient indicator of jump-function recovery. Especially during this period, assessment is needed not only of the ability to perform the test but also of the manner of its performance and the quality of load control.

The improvement achieved in the period of 9–12 months requires additional analysis. Edwards et al. showed that the reduction in CMJ asymmetry after ACLR using HT may result partly from a decrease in values on the non-operated side, not solely from improvement of the operated limb [33]. This indicates that normalisation of the LSI may not always reflect real recovery of mechanical performance. A similar problem concerns patients who meet the symmetry criterion in one test but still present abnormal biomechanics in other dynamic tasks [48,69].

In long-term follow-up, the functional picture remains varied. Jordan et al. indicated that asymmetry indices may normalise approximately 2 years after ACLR, while jump height and peak mechanical power may remain reduced for up to 5 years [39]. Other studies suggest the persistence of compensatory strategies or high LSI values in selected long-term observations, which nevertheless does not determine full normalisation of all biomechanical parameters [47,65]. Taken together, these results indicate that the temporal course of vertical jump performance recovery after ACLR should be interpreted multidimensionally, taking into account not only limb symmetry but also absolute values and assessment of the quality of task performance [33,39,48,71].

### 4.3. Functional Testing and Asymmetry Interpretation During Vertical Jump Tasks

Interpretation of vertical jump results after ACLR requires consideration of the specifics of the test used, because individual tasks assess different components of neuromuscular function. The bilateral CMJ allows analysis of the ability to generate and absorb force under dynamic conditions but at the same time enables compensation by the non-operated limb. Therefore, interpretation of jump height alone, without analysis of the contribution of each limb, may lead to underestimation of loading asymmetry. Studies indicate that post-ACLR deficits may primarily concern impulse asymmetry and limb-loading strategies, even when the global task result appears satisfactory [8,32,33,37,47,48,49]. The work of Kotsifaki et al. [70] is of similar importance; it showed that in athletes assessed at the return-to-sport stage, asymmetries may persist in various vertical jump tests, especially in the concentric phase during landing [70].

Single-leg tests, such as the SL CMJ and SL VJ, better reveal deficits of the operated limb that may be partly masked in bilateral tasks [34,58]. Their interpretation should not, however, be based solely on the LSI ≥ 90% threshold, because the symmetry index does not inform about absolute values, the quality of the movement strategy or whether the non-operated limb has retained its level of fitness [33,48,72]. In this context, the observations of Chen et al. are important; they showed that patients meeting the symmetry criterion in a single-leg test may still present abnormal biomechanics during the bilateral vertical jump [48].

The DVJ and DJ tests provide information on landing control, load absorption and stabilisation of the limb in the frontal plane. In this review, they were particularly useful for assessing dynamic knee valgus and landing strategies after ACLR [23,36,46,52,53,60,62,64]. In turn, the SJ, by limiting the contribution of the countermovement, better isolates the concentric ability to generate force. The results of Pairot de Fontenay et al. indicate that deficits during the single-leg SJ may result not only from knee joint limitations but also from compensation within the hip and ankle [51].

These data indicate that a single test or a single index, especially the LSI alone, is insufficient for full interpretation of vertical jump function after ACLR. It is more justified to use a battery of tests with complementary diagnostic value: the CMJ allows assessment of the global ability to generate and absorb force and of the contribution of individual limbs; single-leg tests better expose deficits of the operated limb; the DVJ and DJ enable analysis of landing quality; and the SJ provides information on concentric force generation. Such a strategy allows better differentiation of real functional recovery from apparent normalisation of symmetry and identification of persisting movement compensations after ACLR [48,69,71,73].

### 4.4. Practical Implications

The results of this review indicate that graft type after ACLR should be taken into account in the interpretation of postoperative vertical jump performance but should not be treated as an independent determinant of functional fitness. In practice, the most important aspect is linking information about the graft type with the objectively determined deficit profile. In patients after BPTB, particular attention may be required for control of the eccentric phase and load tolerance of the extensor apparatus, whereas for HT and QT, the assessment should comprise both the ability to generate power and the quality of landing control [5,8,40,50,52].

Assessment of vertical jump performance after ACLR should not be based on a single test or on jump height alone. It is more clinically useful to use a battery of tests comprising bilateral and single-leg tasks and landing tests, supplemented by analysis of kinetic and kinematic parameters and of the quality of movement performance [32,33,34,48,51,58]. Such an approach allows better differentiation of force-generation deficits and compensatory strategies.

In the context of decisions on load progression and return to sport, the way in which the LSI is interpreted becomes particularly important. Although the LSI ≥ 90% threshold remains clinically useful, it should not be treated as an independent criterion for recovery of vertical jump function or readiness to return to sport, because it may mask persisting deficits of power or landing quality [33,48,69,72]. Decisions regarding load progression and return to sport should therefore be based on a multidimensional battery of tests, comprising not only limb symmetry but also absolute values, landing control, time from surgery and the patient’s sport-specific requirements [74].

The practical implications outlined above must be clearly separated from the evidence directly examined in this review. This review evaluated vertical jump performance parameters only; it did not analyse clinical outcomes or the effectiveness of any rehabilitation protocol. The evidence assembled here therefore does not support recommendations regarding graft selection or return-to-sport clearance. The statements in this section represent interpretative guidance derived from general principles of functional assessment.

### 4.5. Potential Confounding Factors Influencing Functional Recovery

Vertical jump performance after ACLR depends on many variables, among which graft type is probably one of the less important. The included studies did not report the type, frequency or load progression of rehabilitation in a standardised manner, and in only some was this programme described at all. Because both jump height and asymmetry indices respond strongly to strength and plyometric training, differences attributed to graft type may reflect differences in rehabilitation. This is illustrated by the observation that targeted eccentric training in the late phase increased CMJ height irrespective of graft type [28].

In the largest of the included studies, demographic factors were stronger determinants of outcome than graft type [50], and psychological readiness was associated with return to sport independently of functional ability [2,38]. Jump height calculated from flight time and from force impulse are not interchangeable quantities, so some discrepancies may be purely methodological; it has also been shown that repeated test administration produces learning effects in coordinative tasks [54]. Taken together, these factors mean that graft type should not be treated as an independent explanatory variable for vertical jump performance, and the discrepancies between studies probably stem to a greater extent from the uneven distribution of these variables than from biological differences between grafts.

### 4.6. Limitations and Future Recommendations

This review has several limitations that should be taken into account when interpreting the results. The most important is the substantial heterogeneity of the included studies, comprising differences in study designs and in population characteristics. For this reason, a full meta-analysis covering all studies was not possible, and the synthesis was mainly narrative, in accordance with the SWiM assumptions. An additional limitation was the uneven number of studies for the individual graft types. Most data concerned HT and BPTB, whereas evidence on QT and allografts was less numerous and more methodologically varied.

Future studies should focus on prospective, direct comparisons of the most commonly used grafts, especially BPTB, HT and QT, using standardised vertical jump assessment protocols. Studies with clearly defined observation time points, comprising the early, intermediate and long-term period after ACLR, are particularly needed. This would make it possible to better determine whether graft-dependent differences observed in the first months of rehabilitation persist, equalise or transform into different compensatory strategies.

Further limitations include the restriction of the search to English-language, peer-reviewed publications. Formal assessment of publication bias was not possible, as graphical and statistical methods require quantitative synthesis. Some included studies may involve partially overlapping patient cohorts (Section 3.2), which could inflate the apparent number of independent observations. Vertical jump performance itself is only an indirect marker of readiness for sport; none of the included studies validated the analysed parameters against the incidence of ACL re-injury, and no conclusions regarding the safety of return to sport can be drawn from this review.

Greater standardisation of results reporting is also necessary. Future work should take into account not only jump height and LSI but also absolute values, eccentric and concentric impulse, peak power, ground reaction force, joint work, landing biomechanics and dynamic knee valgus. The CMJ, SL CMJ/SL VJ, DVJ/DJ and SJ should not be treated as equivalent endpoints; therefore, future studies should use test batteries comprising bilateral and single-leg tasks and landing tests.

An important direction for further research is also to determine which vertical jump parameters best predict safe return to sport, the risk of re-injury and the long-term functional outcome. Instead of basing decisions solely on the LSI ≥ 90% threshold, future studies should analyse combinations of multiple indices. Such an approach could facilitate the creation of more individualised rehabilitation strategies dependent on graft type and return-to-sport decisions.

## 5. Conclusions

This systematic review included 47 studies involving more than 3400 participants. The certainty of evidence was rated as low for one clinical question and very low for all other questions; therefore, the following conclusions should be considered preliminary. The available evidence does not indicate that graft type determines vertical jump height after anterior cruciate ligament reconstruction (ACLR). An association was observed suggesting that eccentric-phase asymmetry during the bilateral countermovement jump (CMJ) may be greater following bone–patellar-tendon–bone (BPTB) graft reconstruction than following hamstring tendon (HT) graft reconstruction during the early and intermediate stages of rehabilitation. However, this association was inconsistent across studies. Comparisons between autografts and allografts, which represented the most consistent body of evidence in this review, showed no differences in vertical jump performance. Clinical decisions regarding graft selection should therefore be based on established clinical criteria. Vertical jump deficits may persist for many months and, in some cases, for several years after surgery. Symmetry indices and absolute values appear to normalise at different rates. Because most of the available data are cross-sectional, a consistent recovery trajectory cannot be established, nor can clear thresholds for clinical decision-making be identified. Assessment of vertical jump function after anterior cruciate ligament reconstruction (ACLR) should include symmetry indices, absolute values and the quality of landing control. An LSI ≥ 90% should not be used as a stand-alone return-to-sport criterion. This review provides no basis for recommendations regarding graft selection or for modifying rehabilitation protocols according to graft type. Establishing such relationships requires prospective randomised trials using standardised assessment protocols and consistent time points. The choice between autograft and allograft does not significantly affect vertical jump performance after ACLR. The clinical decision on graft type should therefore be based on other clinical criteria, such as joint stability, the risk of reoperation and patient preferences.

The temporal course of deficits after ACLR is biphasic: symmetry indices normalise approximately 2 years after surgery; however, other values, such as, for example, jump height or peak mechanical power, may remain reduced for up to 5 years after surgery, and full recovery of vertical jump symmetry is achieved only at 5–9-year follow-up.

From a clinical perspective, intensification of eccentric work in the rehabilitation protocol is recommended in patients after a BPTB graft in the first 9 months after surgery, together with consideration of a QT graft in high-risk patients because of the more favourable dynamic valgus profile.

## Data Availability

The article was registered in the PROSPERO database and is publicly available under the number CRD420261386307.

## Supplementary Materials

The following supporting information can be downloaded at: https://www.mdpi.com/article/10.3390/jcm15176588/s1.
