# Supplementary material for: The Effect of Graft Type on Vertical Jump Performance After Anterior Cruciate Ligament Reconstruction: A Systematic Review
Source: J Clin Med. 2026 Aug 26;15(17):6588. doi: 10.3390/jcm15176588 (PMC13566778; doi:10.3390/jcm15176588)
Supplement: Supplementary file 1 [file jcm-15-06588-s001.zip › Supplementary File S1.pdf]

A. STUDIES WITH A BPTB GRAFT (Bone-Patellar Tendon-Bone) – AUTOGRAFT ONLY												
No.	Author, Year	n	Age	Sex	Graft	Time post – op	Jump test	Outcome	Involved limb	Uninvolved/Control	LSI / AI (%)	Key Findings
1	Bach 1998	n=97	28.8	F+M	BPTB auto	60–108 (5–9 years)	single-leg VJ	S-S asym. (%)	VJ height comparable to the healthy side; asymmetry <2%	VJ height of the uninvolved limb (reference) – 100%	LSI 98%	Very good vertical-jump symmetry 5–9 years after BPTB. Side-to-side deficit <2%. Full functional recovery at long-term follow-up.
2	Baumgart 2015	n=50	29.2±8.5	F+M	BPTB auto	31 ± 7 (2.5 years)	CMJ 2- and 1-leg	GRF	Lower ground reaction force (GRF) than on the healthy side; asymmetries dependent on the IKDC score	Higher GRF values on the healthy side (reference limb)	Dependent on the IKDC score	At 2.5 years after BPTB, patients show 3 compensatory GRF strategies. The lower the IKDC score, the greater the inter-limb asymmetry.
3	Bayrakci 2008	n=37	25.2±6.5	M	BPTB auto	6	single-leg VJ	VJ height	Significantly lower vertical-jump height ($p<0.05$)	Higher jump height on the healthy side (reference)	75% of quadriceps strength on the operated vs non-operated side	At 6 months after BPTB, vertical-jump height is lower on the operated side. Quadriceps strength operated/non-operated = 75%;

4	Fontenay 2014	n=13 ACLR n=16 C	27.2±3.8	M	BPTB auto	6–9	single-leg SJ	WTotal, Hflight	Flight-phase height (Hflight) = 65% of the healthy side; total joint work (WTotal) = 76%	Healthy control group: Hflight = 89%; WTotal = 99% (symmetry norm)	Hflight 65% vs 89% ($p<0.001$)	At 6–9 months after BPTB, asymmetries occur in single-leg SJ height (35% deficit) and total work (24% deficit). Values differ significantly from the control group ($p<0.001$).
5	O'Malley 2018	n=118 ACLR n=44 C	24.4±4.1	M	BPTB auto	≥6	SL CMJ	Height, LSI, power	Lower SL CMJ height than in the healthy group (Cohen's $d > 0.4$); LSI significantly reduced ($d > 1.1$)	Healthy control group: higher values of jump height, peak power and LSI (reference)	LSI and ISO $d>1.1$ vs C	At ≥6 months after BPTB, SL CMJ height and LSI were reduced vs healthy controls.
6	Petschnig 1998	n=50 ACLR n=16 C	28.2±9.1	F+M	BPTB auto	11.7 ± 5.9	single-leg VJ	VJ height (cm) PT–Q correlation	Significantly lower single-leg VJ height than on the healthy side; strong correlation with quadriceps strength ($r=0.51$, $p<0.001$)	Single-leg VJ height on the non-operated side within the normal range in the control group	VJ lower operated vs non-operated	At 12 months after BPTB, single-leg vertical jump was lower on the operated side. Strong correlation with quadriceps strength ($r=0.51$).

B. STUDIES WITH AN HT GRAFT (Hamstring Tendon) – AUTOGRAFT ONLY

No.	Author, Year	n	Age	Sex	Graft	Time post-op (months)	Jump test	Outcome parameter	Limb outcome operated	Limb outcome healthy/control	LSI / AI (%)	Key findings

1	Ebert 2021	n=50	28.3±9.1	F+M	HT auto	10.2 ± 1.4	SLCMJ	LSI SLCMJ (%)	Mean SLCMJ LSI = 83.4 ± 9.5%	SLCMJ height of the uninvolved limb (reference) – 100%	LSI: 83.4±9.5% (lowest)	At 10 months after surgery, only 18% of patients reached the clinically satisfactory LSI ≥90% threshold.
2	Edwards 2025	n=91	29.5±9.5	F+M	HT auto	6 and 9–12	CMJ + DJ	Height, RSI, AI	CMJ height ↑1.46 cm; DJ height ↑2.35 cm (improvement T1→T2)	Decline in force impulse on the non-operated side over time	AI con: ↓3.13% AI ecc: ↓3.59%	After reconstruction, CMJ and DJ jump height improves at 6→9–12 months. However, the reduction in asymmetry results mainly from a decline in the performance of the healthy limb rather than from improvement of the operated one.
3	Forelli 2025	n=64 ACLR n=47 C	26.5±5.0	F+M	HT auto	6	CMJ 2-leg	LSI vGRF, MP, RFDe	LSI vGRF = 85.9%; jump height = 21.1 ± 7.4 cm	Healthy control group: LSI vGRF = 94.9%; jump height = 28.7 ± 10.0 cm	vGRF: 85.9 vs 94.9% (p<0.001)	At 6 months after grafting, the braking phase in the CMJ was impaired (p<0.001). The greatest deficit concerned the eccentric phase; jump height was 26% lower than in healthy controls.
4	Ghorbani 2022	n=12 ACLR n=12 C	25.5±3.7	24M	HT auto	6–24	Jump + landing	TTS	Time to Stability (TTS) comparable to the healthy group before and after the protocol	Healthy control group: TTS comparable to ACLR (no significant differences)	No significant differences	No differences in time to stabilization (TTS) under plyometric fatigue between individuals 6–24 months after ACL reconstruction and uninjured participants.

5	Krafft 2020	n=20 ACLR + control	27.8±8.2	F+M	HT (ST+G)	0 / 1.75 / 3 / 6 (preop --> 6)	CMJ 2-leg	Angles, moments of knee extension	Maximum knee-extension moments increase from preoperative values to 6 months postoperatively but remain lower than in the healthy group	Healthy control group: symmetric, higher knee-extension moment values (reference)	Deficit persists at 6m vs C	After surgery, CMJ kinematics and kinetics improve gradually up to 6 months but do not reach the level of healthy individuals.
6	Kurz 2022	n=17 ACLR n=67 C	ACLR: 25±7 C: 23±4	F+M	HT auto	11 (6–23)	SJ + DJ single-leg	SJ power, DJ TOE, LSI	Lower single-leg SJ peak power and lower DJ rebound efficiency than in the healthy group	Healthy control group: higher SJ power and DJ TOE values (reference)	SJ LSI correlates with the outcome (r=0.46)	At 11 months after grafting, despite clinical return to sport, SJ peak power and DJ rebound efficiency remain lower than in healthy controls.
7	Legnani 2023	n=51	30.1±11.2	F+M	HT auto	6	CMJ 1/2-leg	LSI, RSI	CMJ jump height reduced on the operated side; LSI below reference norms ($p<0.05$)	CMJ jump height of the non-operated limb (within-patient reference)	LSI < norm ($p<0.05$)	At 6 months after HT, jump LSI was reduced.
8	Legnani 2024	n=33	34.0±11.5	F+M	HT auto	6 and 12	SJ, CMJ 2/1-leg	Jump height, LSI	Single-leg CMJ height increases significantly from 6 to 12 months ($p<0.01$); asymmetry persists at 6 months.	CMJ height of the non-operated limb (within-patient reference)	Asymmetry 6m; improvement by 12m	After grafting, single-leg CMJ jump height improves significantly between 6 and 12 months. Asymmetries persist at 6 months, but LSI does not correlate with achieving pre-injury-level RTS.

9	Moya-Angeler 2017	n=74	34.0 ±9	74M	HT (ST+G)	0 / 3 / 6 / 12 (preop --> 12)	DVJ, VJ, SL hop (FP)	LSI (%)	Bilateral tests (DVJ, VJ): LSI <90%; single-leg tests (SL hop): LSI >90% – differing sensitivity	Jump height of the non-operated limb (within-patient reference)	DVJ/VJ <90%; SL hop >90%	Bilateral tests (DVJ, VJ) detect asymmetries better than single-leg hop-type tests
10	Ohji 2024	n=65	21.0±5.0	F+M	HT auto	13 (median)	SL VJ + SL DJ	LSI SL VJ, RSI DJ	Patients not achieving RTS: lower SL VJ LSI and lower SL DJ RSI than those achieving RTS (p<0.05)	Jump height of the non-operated limb (within-patient reference)	LSI SL VJ: No-RTS < Yes-RTS	At 13 months after grafting, single-leg vertical-jump LSI (SL VJ) and drop-jump RSI differentiate patients who achieve return to sport from those who do not (p<0.05).
11	Gobbi 2003	n=80	28	F+M	ST (quadrupl.)	36 (24–52)	single-leg VJ + hop	VJ and hop	Single-leg VJ height within the non-operated side's normal range already from 6 months; full isokinetic strength from 12 months	VJ height of the uninvolved limb (reference) – 100%	Normal from 6m;<br>PT normal 12m	After grafting, vertical-jump height normalizes quickly (from 6 months); full quadriceps strength from 12 months.
12	Ortiz 2014	n=14 ACLR n=16 C	25.4±5.2	F	ST+G auto	12–60 (1–5 years)	DJ 1/2-leg	Dynamic valgus, EMG	Tendency toward greater dynamic knee valgus during the DJ than in healthy controls (n.s.)	Healthy female control group: smaller dynamic valgus during the DJ	Different EMG strategies no signif. kinematic differences	1–5 years after grafting, participants retain a tendency toward greater dynamic valgus during the drop jump. Different EMG activation strategies suggest compensation at the neuromuscular level.

13	Ueda 2019	n=420	23.1±8.9	F+M	HT auto	0 / 3 / 6 / 9 / 12 (preop --> 12)	1-leg hop	LSI hop, QSI	Patients in the "poor" group: preoperative quadriceps strength deficit: lowest LSI and greatest fear of jumping	Jump height of the non-operated limb (within-patient reference)	LSI dependent on preoperative quadriceps strength	After HT, the preoperative quadriceps strength deficit determines the rate of LSI recovery on the single-leg hop and the level of kinesiophobic fear. The preoperative status is prognostic for the final outcome.
14	Çağlayan 2025	n=55	ACLR: 34±4 C: 21±2.94	M	HT auto	≥3 (≥12 weeks)	VJ + hop + Y-Balance	LSI VJ (%)	Intervention group (multimodal RTS training): significantly greater improvement in VJ LSI than the standard group ($p<0.05$)	ACLR control group (standard rehabilitation): smaller LSI improvement	VJ LSI: greater improvement in interv. group	At ≥3 months after HT, multimodal RTS training improves vertical-jump and hop-test LSI significantly more effectively than standard rehabilitation ($p<0.05$).
15	Mioc 2025	n=68	ACLR: 22.21±5.34; ACLR-LET: 18.9±4.76	F+M	HT auto ± LET	6–8	DJ 2- and 1-leg	RSI, LSI Q	Quadriceps strength and drop-jump RSI comparable between groups with and without LET	Jump height of the non-operated limb (within-patient reference)	Quadriceps LSI similar both groups	At 6–8 months after HT, adding lateral extra-articular tenodesis (LET) did not significantly affect quadriceps strength or drop-jump RSI.
16	Larsen 2014	n=23	27.2±7.5	M	HT auto	27 ± 7 (2.25 years)	CMJ 2-leg	Kinematics, MVC, OLJD	75% of patients show a quadriceps strength deficit >15%; OLJD and MVC correlate with the KOOS score	Jump height of the non-operated limb (within-patient reference)	Correlations with KOOS (r^2=0.26–0.53)	At 2 years after grafting, quadriceps strength deficits and OLJD explain 31–53% of the variance in subjective knee assessment (KOOS). CMJ kinematics adds little additional explanatory value.

17	Jahnke 2025	n=32	31.7±8.5	F+M	HT (ST) auto	6 (±1)	SL CMJ	SL CMJ height; learning effect (Set 1 vs Set 3)	SL CMJ height 0.08→0.06 m (Set 1→Set 3; −25%)	Non-operated limb: 0.10→0.11 m (+10%; p=0.657)	Operated < non-operated (height 0.06–0.08 vs 0.10–0.11 m)	At 6 months after ST autograft, SL CMJ height of the operated limb was 25% lower than the non-operated limb.

C. COMPARATIVE STUDIES: BPTB vs HT (direct comparison)

No.	Author, Year	n	Age	Sex	Graft	Time post-op (months)	Jump test	Outcome parameter	Limb outcome operated	Limb outcome healthy/control	LSI / AI (%)	Key findings
1	Costley 2021	n=44 (HT=22, BPTB=22)	HT:26.1 BPTB:23.4	M	HT vs BPTB	6 and 9	CMJ 2-leg	Phasic impulse (ecc/con) asym.	CMJ impulse asymmetries persist at 6–9 months; graft type does not modify the outcome (p>0.05)	Jump height of the non-operated limb declines over time (possible detraining effect)	Con asym.↓; Ecc persists	At 6–9 months, both BPTB and HT show persistent asymmetries in the eccentric phase of the CMJ. Graft type does NOT significantly affect the results (p>0.05).
2	Miles 2019	n=66 (BPTB=22,HT=22,C=22)	BPTB:23.4 HT:25.6	M	BPTB vs HT	9	CMJ 2-leg	Impulse ecc/con asym.	BPTB: significantly higher eccentric impulse asymmetry than HT (p=0.008)	Healthy control group: symmetric CMJ impulse between limbs (reference)	BPTB > HT asym. ecc and con	At 9 months after ACLR, the BPTB graft produces greater eccentric-phase CMJ asymmetry than HT (p=0.008). Quadriceps strength is a predictor of concentric-phase asymmetry.

3	Labanca 2022	n=265 (STGR=119,BPTB=146) C=24	27	F+M	BPTB vs STGR	3 and 6–9	CMJ 2-leg	Peak force, power, LSI	T1 (3 months): BPTB has higher peak power but lower LSI than STGR. T2 (6–9 months): no LSI differences	Healthy control group: higher LSI than both ACLR groups (reference)	T1: LSI BPTB<STGR T2: equalization	BPTB yields higher peak CMJ power but greater asymmetries at 3 months postoperatively than STGR. By 6–9 months the differences between grafts disappear, although both groups still have lower LSI than healthy controls.
4	Jordan 2022b	n=34 ACLR (ST=29,BPTB=5) C=178	elite athletes	F+M	ST vs BPTB	2–24	CMJ 2-leg + isokinetics	AI ecc/con CMJ, AI MVC, RTD	Eccentric AI: 13% (<6 months) --> 8% (>12 months); BPTB has significantly higher AI than ST (p<0.05)	Healthy athlete control group: AI = 5 ± 5% (symmetry reference)	AI ecc CMJ: 13-->8%; AI con: 14-->6%	BPTB produces significantly higher CMJ asymmetries than ST throughout the rehabilitation period (p<0.05). The GAMM model predicts a slower return to symmetry after BPTB.
5	Papalia 2014	n=40 (HT=20, BPTB=20)	HT:29.6 BPTB:30.2	F	HT vs BPTB	3 and 6	DVJ	KAM	Knee valgus moment (KAM) improves from 3 to 6 months in both groups; BPTB reaches the physiological norm faster than HT	Preoperative values as reference	BPTB+propriocep.: KAM within norm at 6m	At 6 months, BPTB combined with proprioception-focused rehabilitation yields significantly better dynamic-valgus values, which are smaller than with HT (p<0.0001).
6	Pasquini 2017	n=30 (BPTB=15,HT=15)	BPTB:26.8 HT:28.4	M	BPTB vs HT	12	SJ	SJ height (cm)	BPTB: significantly higher operated-limb SJ height than HT (p=0.037)	SJ height of the non-operated limb (within-patient reference)	BPTB > HT height SJ operated	At 12 months, BPTB yields higher operated-limb squat-jump height than HT (p=0.037). Opposite direction to the CMJ (where BPTB performed worse). No differences in stabilometry.

7	Peebles 2021	n=38 (BPTB=19,HT=19)	16.3±1.9	F+M	BPTB vs HT	6 (25.7 ± 6.2 weeks)	Stop-jump landing	LSI of moment knee ext.	BPTB: greater knee-extension moment asymmetry than HT during the stop-jump (p=0.006)	Jump height of the non-operated limb (within-patient reference)	36.9% of variance LSI explained	At 6 months after grafting, BPTB produces significantly greater knee-extension moment asymmetry during landing than HT (p=0.006). Psychological readiness is a predictor of asymmetry (r=0.325).
8	Andrade 2023	n=25 (HT=8,BPTB=6,C=11)	18-45	F+M	HT vs BPTB	6–72	DVJ	vGRF, kinematics	Lower vGRF than in the healthy group (p=0.049); no valgus differences between HT and BPTB	Healthy control group: higher vGRF – 20.11 ± 6.29 N/kg (reference)	Not reported jump LSI	At 6–72 months after ACLR, vGRF is significantly lower than in healthy controls (p=0.049). No kinematic differences between HT and BPTB in the DVJ test; graft type does not modify valgus.
9	Torre 2024	n=44	27.5±6.9	F+M	BPTB vs HT	3	CMJ, SL hop, Triple hop	LSI CMJ, SLH, TH (%)	LSI CMJ = 85.3 ± 8.9%; LSI SLH = 92.2 ± 6.4%; no differences BPTB vs HT (p=0.56)	Jump height of the non-operated limb (within-patient reference)	LSI CMJ: 85.3% (lowest)	At 3 months in professional athletes, no significant CMJ LSI differences between BPTB and HT (p=0.56). The CMJ is the most sensitive test.

D. STUDIES WITH A QT GRAFT (Quadriceps Tendon) AND QT vs HT/ST COMPARISONS												
**No.**	**Author, Year**	**n**	**Age**	**Sex**	**Graft**	**Time post-op (months)**	**Jump test**	**Outcome parameter**	**Limb outcome operated**	**Limb outcome healthy/control**	**LSI / AI (%)**	**Key findings**

1	Csapo 2020	n=245 (QT=139,HT=88)	23.8±8.4	F+M	QT vs HT	6 (185 ± 44 days)	SL CMJ	SL CMJ height, BIA score	24.5% of patients with SL SJ failure of the operated limb; 47.3% with ≥1 'weak' test; no differences QT vs HT	Population norms by age and sex (BIA database)	47.3% ≥1 test weak	At 6 months, no significant vertical-jump differences QT vs HT (p>0.05). Deficits are more frequent in patients >19 years and in men – demographic factors more important than graft type.
2	Hunnicutt 2024	n=24 (QT=14,PT=10)	QT:25.5 PT:22.3	F+M	QT vs PT	6–24	DVJ	Peak vGRF	Peak vGRF: QT operated = 1.10 N/kg body mass; PT operated = 1.10 N/kg (identical)	Peak vGRF of the non-operated limb: QT = 1.30 N/kg; PT = 1.35 N/kg (within-patient reference)	Both groups lower operated (p<0.05)	At 6–24 months, no biomechanical DVJ differences between QT and BPTB (p>0.05). Both groups unload the operated limb in a similar way.
3	Gaugg 2026a	n=26 (ST=15,QT=11)	M:28.9 F:24.0	F+M	ST vs QT	8–9	VDJ	Valgus angle	Women: greater contralateral knee valgus than men (p=0.016); no differences ST vs QT in the hop test	Men with ACLR: smaller contralateral valgus	No LSI differences hop ST vs QT	At 8–9 months, women show greater valgus regardless of graft (p=0.016). No differences ST vs QT in hop-type testing.
4	Gaugg 2025	n=26 (ST=15,QT=11)	27.4±6.1	F+M	ST vs QT	8	VDJ	Max valgus angle (°)	ST: valgus angle −2.4° (valgus); QT: +3.5° (varus) – significant difference (p=0.027)	No significant angular differences between the operated and contralateral limb in either group	ST vs QT difference (p=0.027)	At 8 months, the QT graft yields significantly smaller dynamic valgus than ST (p=0.027).

E. STUDIES WITH ALLOGRAFTS AND AUTOGRAFT vs ALLOGRAFT COMPARISONS

No.	Author, Year	n	Age	Sex	Graft	Time post-op (months)	Jump test	Outcome parameter	Limb outcome operated	Limb outcome healthy/control	LSI / AI (%)	Key findings
1	Sun 2009	n=156 (auto=76,allo=80)	28	F+M	BPTB auto vs BPTB allo	67,2 (56–102; 5.6 years)	VJ Harner + hop	LSI VJ (%)	VJ height comparable in both groups (BPTB auto = BPTB allo); no differences in VJ LSI or hop LSI	Jump height of the non-operated limb (within-patient reference)	VJ LSI: none differences	At 5.6 years, BPTB autograft and allograft yield equivalent vertical-jump results (p>0.05). Allograft is a good alternative to autograft.
2	Sun 2011a	n=69 (non-ir=38,ir=31)	27	F+M	HT allo irrad. vs non-irrad.	41	VJ Harner + hop	LSI VJ (%)	VJ height comparable in both groups (irradiated vs non-irradiated); joint stability worse in the irradiated group	Jump height of the non-operated limb (within-patient reference)	VJ LSI: no differences	At 3.5 years, irradiation of the HT allograft does not affect vertical-jump height, even though it worsens knee joint stability.
3	Sun 2011b	n=166 (auto=85,allo=81)	27-28	F+M	HT auto vs HT allo	67 (5.6 years)	VJ Harner + hop	LSI VJ (%)	VJ height comparable in both groups (HT auto = HT fresh-frozen allo)	Jump height of the non-operated limb (within-patient reference)	LSI VJ: no differences	At 5.6 years, HT autograft and fresh-frozen allograft yield equivalent vertical-jump results (p>0.05). No functional preference between graft types.
4	Tian 2016	n=87 (auto=43,ir-allo=44)	27	F+M	HT auto vs HT irrad. allo (DB)	59 (5 years)	VJ Harner + hop	LSI VJ (%)	VJ height comparable in both groups (HT auto = HT irradiated allo, DB technique; p=0.677)	Jump height of the non-operated limb (within-patient reference)	VJ LSI: no differences	At 5 years with the double-bundle technique, vertical jump does NOT differ significantly between HT autograft and irradiated allograft (p=0.677).

5	Fox 2004	n=32 revision	28 (16-57)	F+M	BPTB allo (revision)	57,6 (25–145)	single-leg VJ	S-S asym. (height)	Mean VJ height = 98% of the non-operated side, but high variability (SD 33.2%; range 35–232%)	Jump height of the non-operated limb (reference) – 100%	VJ LSI: 98% (SD=33%)	In BPTB allograft revisions, mean VJ symmetry is good, but the variability of results is very high (SD 33%).
6	Oeffinger 2000	n=23 (allo=7,auto=8,C=8)	22-27	F+M	BPTB auto vs BPTB allo	auto: 27.6 ± 10<br>allo: 21.3 ± 5	single-leg VJ	VJ height (cm)	VJ height: BPTB auto = 38.5 ± 5 cm; BPTB allo = 36.8 ± 6 cm	Healthy control group: VJ height = 39.9 ± 6 cm (reference)	VJ: no differences (p=0.47)	At 25 months, no VJ height differences between BPTB autograft and allograft (p=0.47); both groups within the normal range relative to controls.

F. STUDIES WITH MIXED/UNSPECIFIED GRAFTS												
**No.**	**Author, Year**	**n**	**Age**	**Sex**	**Graft**	**Time post-op (months)**	**Jump test**	**Outcome parameter**	**Limb outcome operated**	**Limb outcome healthy/control**	**LSI / AI (%)**	**Key findings**
1	Chen 2024	n=70 (ACLR=46,C=24)	22-28	F+M	BPTB lub HT (combined)	15,8 ± 5,1	CMJ 2-leg + SL VJ	Kinematics/ CMJ kinetics	Lower knee-extension moments than on the healthy side and than in the control group	Healthy control group: higher knee-extension moments and vGRF	SL VJ LSI: <90%=23; >90%=23	At 16 months after ACLR, even patients with LSI ≥90% on the SL VJ still show abnormal CMJ biomechanics.

2	Jordan 2017	n=22 (ACLR=11,C=11)	ACLR:22±4	F+M	ST(7),BPTB(1) Allo (3)	39.3 ± 32.6 (3.3 years)	SJ powtarzany	AI, EMG, jump height	AI in the propulsion phase significantly elevated; no change after the fatigue protocol	Healthy control group: symmetric AI values	AI ACLR > Control (p<0.001)	At 3.3 years, individuals after ACLR show elevated SJ asymmetry in the propulsion phase (p<0.001), independent of fatigue.
3	Jordan 2022a	n=41 women (ACLR=17,C=30)	16-32	F	ST auto	up to 60 (up to 5 years)	CMJ 2-leg	JH, PP, RSImod, AI	JH height and peak power (PP) significantly reduced up to 5 years after ST (p<0.01)	Healthy control group: values increasing with age	AI ecc: 3±7% AI con: 1±5%	AI normalizes after 2 years, but absolute stretch-shortening cycle values (JH, PP) remain reduced up to 5 years.
4	Stojanovic 2023	n=16	25.5±3.7	M	Not specified	6–12	CMJ, SL VJ, hop	CMJ height, SLJ LSI	CMJ height ↑ after eccentric training (ES=2.23); SL hop LSI ↑ significantly	Values of both limbs improve after the intervention	SLJ LSI ↑ after eccentric training	At 6–12 months after ACLR, eccentric training in late rehabilitation significantly improved CMJ height (large effect ES=2.23).
5	Xue 2024	n=48 (ACLR=32,C=16)	25-26	F+M	Not specified	12–36	DVJ	EMG, kinem./kinet. DVJ	Lower activation of the vastus medialis, rectus femoris and vastus lateralis muscles of the operated limb than in the control group	Healthy control group: higher quadriceps activation (reference)	EMG deficits ACLR vs C	At 12–36 months after ACLR, preoperative limb dominance influences the EMG asymmetry pattern during the DVJ, which is a prognostic factor.

LEGEND:

GRAFTS:

BPTB – Bone-Patellar Tendon-Bone (bone–patellar tendon–bone)

HT – Hamstring Tendon (hamstring muscle tendons: semitendinosus ± gracilis)

ST – Semitendinosus (semitendinosus muscle tendon)

QT – Quadriceps Tendon (quadriceps femoris tendon)

STGR – Semitendinosus + Gracilis (semitendinosus and gracilis muscle tendons)

LET – Lateral Extra-articular Tenodesis

auto / allo – autograft / allograft

ir / non-ir – irradiated / non-irradiated; DB – double-bundle

VERTICAL-JUMP TESTS:

CMJ – Countermovement Jump

SJ – Squat Jump (from a squat, without countermovement)

DVJ / VDJ – Drop Vertical Jump (vertical jump following a drop from a raised platform)

DJ – Drop Jump (drop landing with immediate rebound)

SL CMJ / SL VJ – Single-Leg CMJ / Vertical Jump (single-leg vertical jump)

VJ – Vertical Jump; VJ Harner – jump test according to the Harner protocol

BIA – Back in Action (standardized battery of functional tests)

FP – Force Plate; dual FP – dual force plates

OUTCOME PARAMETERS:

LSI – Limb Symmetry Index = (operated / non-operated limb) × 100%

AI – Asymmetry Index (inter-limb asymmetry index)

RSI – Reactive Strength Index (jump height / ground contact time)

RSImod – Modified RSI (jump height / contraction time)

vGRF – Vertical Ground Reaction Force

PP – Peak Power (peak mechanical power)

RFD / RFDe – Rate of Force Development / eccentric

KAM – Knee Abduction Moment (knee abduction/valgus moment)

SSC – Stretch-Shortening Cycle

JH – Jump Height; TTS – Time to Stability

TOE – Take-off Efficiency; WTotal – total mechanical joint work

Hflight – flight-phase height; S-S – side-to-side

ecc – eccentric phase; con – concentric phase

OTHER ABBREVIATIONS:

ACLR – anterior cruciate ligament reconstruction

EMG – electromyography
