# Supplementary material for: The Effect of Graft Type on Vertical Jump Performance After Anterior Cruciate Ligament Reconstruction: A Systematic Review
Source: J Clin Med. 2026 Aug 26;15(17):6588. doi: 10.3390/jcm15176588 (PMC13566778; doi:10.3390/jcm15176588)
Supplement: Supplementary file 1 [file jcm-15-06588-s001.zip › Supplementary File S2.pdf]

A. BILATERAL COUNTERMOVEMENT JUMP (CMJ bilateral)						
**Author, Year**	**n**	**Graft**	**Time post-op**	**Jump test**	**Parameter**	**Key findings and interpretation**
Costley 2021	n=44 (HT=22,BPTB=22)	HT vs BPTB	6 and 9	CMJ 2-leg dual FP	Impulse ecc/con	At 6–9 months, eccentric CMJ impulse asymmetries persist in both groups. Graft type (HT vs BPTB) does not modify the outcome ($p>0.05$). Conclusion: braking-phase deficits are common to both graft types.
Miles 2019	n=66 (BPTB=22,HT=22,C=22)	BPTB vs HT	9	CMJ 2-leg dual FP	Impulse ecc/con	At 9 months, BPTB produces a significantly greater eccentric CMJ impulse asymmetry than HT ($p=0.008$). Quadriceps strength is a predictor of concentric-phase asymmetry. Conclusion: BPTB loads the braking phase more than HT.
Labanca 2022	n=265 (STGR=119,BPTB=146)	BPTB vs STGR	3 and 6–9	CMJ 2-leg dual FP	Peak force, PP, LSI	At 3 months, BPTB yields higher peak power (PP) but greater asymmetry than STGR. By 6–9 months the differences between grafts disappear. Conclusion: the advantage of BPTB in power generation is offset over time as the asymmetry equalizes.
Edwards 2025	n=91	HT auto	6 and 9–12	CMJ + DJ dual FP	Height, RSI, AI	CMJ height increases by 1.46 cm between 6 and 9–12 months; concentric-phase AI decreases by 3.13% and eccentric-phase AI by 3.59%. Conclusion: the improvement results mainly from a decline in the performance of the healthy limb rather than from functional recovery of the operated one.

Forelli 2025	n=111 (ACLR=64,C=47)	HT auto	6	CMJ 2-leg dual FP	LSI vGRF, MP, RFDe	At 6 months after HT, LSI vGRF is 85.9% vs 94.9% in healthy controls ($p<0.001$) – a significant eccentric-phase braking deficit. Conclusion: the braking phase in the CMJ is the most impaired after HT and is also clinically more sensitive than the jump-height parameter.
Jordan 2022a	n=41 women (ACLR=17)	ST auto	up to 60 (up to 5 years)	CMJ 2-leg dual FP	JH, PP, RSImod, AI	After ST grafting, jump height (JH) and peak power (PP) remain reduced up to 5 years postoperatively. AI normalizes after 2 years. Conclusion: LSI asymmetry normalizes faster than absolute power values – a risk of false-positive RTS.
Jordan 2022b	n=34 ACLR (ST=29,BPTB=5)	ST vs BPTB	2–24	CMJ 2-leg + isokin.	AI ecc/con, MVC	BPTB produces significantly higher CMJ AI than ST throughout the rehabilitation period ($p<0.05$). Eccentric AI decreases from 13% (<6 months) to 8% (>12 months). Conclusion: the return to symmetry after BPTB is slower.
Krafft 2020	n=20 ACLR + C	HT (ST+G)	0 / 1.75 / 3 / 6 (preop → 6)	CMJ 2-leg 3D+FP	Angles, knee moments	Maximum knee-extension moments in the CMJ increase from the preoperative state to 6 months after HT but do not reach the values of the healthy group. Conclusion: full recovery of knee control during jumping requires >6 months – the time criterion is insufficient for RTS.
Baumgart 2015	n=50	BPTB auto	31 ± 7 (2.5 years)	CMJ 2- and 1-leg	GRF, impulse	At 2.5 years after BPTB, patients show 3 compensatory GRF strategies, also dependent on subjective knee assessment (IKDC). Conclusion: a low IKDC score is associated with greater asymmetry.

Larsen 2014	n=23	HT auto	27 ± 7 (2.25 years)	CMJ 2-leg Vicon	Kinematics, MVC	At 2 years after HT, 75% of patients have a quadriceps strength deficit >15%. Isokinetic strength (MVC) and single-leg jump explain 31–53% of the variance in the KOOS score. Additional conclusion: CMJ kinematics contributes little additional explanatory value – strength tests are key.
Torre 2024	n=44 professionals	BPTB vs HT	3	CMJ + SL hop + TH	LSI CMJ, SLH, TH	At 3 months, LSI CMJ = 85.3% – the lowest result among the functional tests. No differences BPTB vs HT (p=0.56). Additional conclusion: the CMJ is the most sensitive asymmetry test in the early phase after ACLR, more sensitive than hop tests.
Chen 2024	n=70 (ACLR=46,C=24)	BPTB lub HT	15.8 ± 5.1	CMJ 2-leg + SL VJ	Kinematics/kinetics	At 16 months, even in patients with LSI ≥90% on the SL VJ, CMJ biomechanics remains abnormal (lower knee-extension moments). Conclusion: meeting the LSI ≥90% criterion on hop tests does not guarantee full normalization of kinematic parameters.
Stojanovic 2023	n=16	Mixed	6–12	CMJ + SL VJ + hop	CMJ height, SLJ LSI	Eccentric training significantly increases CMJ height (very large effect ES=2.23) and improves LSI on the SL hop. Additional conclusion: progression using eccentrics in late rehabilitation is an effective therapeutic intervention – it justifies inclusion in the RTS protocol.

B. CMJ / SINGLE-LEG VERTICAL JUMP (SL CMJ / SL VJ)						
Author, Year	n	Graft	Time (months)	Jump test	Parameter	Key findings and interpretation

Ebert 2021	n=50	HT auto	10.2 ± 1.4	SLCMJ	LSI SLCMJ (%)	At 10 months after HT, SLCMJ LSI = 83.4 ± 9.5% – the lowest among the 8 hop-type tests. Only 18% of patients reach the LSI ≥90% threshold.
O'Malley 2018	n=162 (ACLR=118,C=44)	BPTB auto	≥ 6	SL CMJ 3D	Height, LSI, power	At ≥6 months after BPTB, SL CMJ height is markedly lower than in healthy controls (Cohen's d > 0.4); LSI reduced (d > 1.1). Additional conclusion: combining the SL CMJ with isokinetic tests best differentiates ACLR patients from healthy controls.
Csapo 2020	n=245 (QT=139,HT=88)	QT vs HT	6	SL CMJ	SL CMJ height	At 6 months, no significant SL CMJ differences between QT and HT (p>0.05). 24.5% of patients show failure on the operated-limb SL SJ test. Conclusion: graft type QT vs HT does not affect single-leg jump outcome – demographic factors are more important.
Legnani 2023	n=51	HT auto	6	CMJ 1/2-leg	LSI, RSI	At 6 months after HT, SL CMJ LSI is significantly reduced and correlates with psychological readiness (ACL-RSI scale). Additional conclusion: psychological status influences motor function.
Legnani 2024	n=33	HT auto	6 and 12	CMJ 1-leg	Jump height, LSI	After HT, SL CMJ height increases significantly between 6 and 12 months (p<0.01). Asymmetry persists up to 6 months. Conclusion: full functional recovery requires >12 months; the 6-month LSI does not predict pre-injury-level RTS.

Ohji 2024	n=65	HT auto	13	SL VJ + SL DJ	LSI SL VJ, RSI	At 13 months after HT, patients not achieving RTS level have a significantly lower SL VJ LSI than those achieving RTS ($p<0.05$). Additional conclusion: SL VJ LSI is a significant predictor of return to sport.
Jahnke 2025	n=32	HT (ST) auto	6 (±1)	SL CMJ	SL CMJ height; learning effect	At 6 months after ST autograft, the SL CMJ shows no significant learning effect between Set 1 and Set 3 (operated 0.08→0.06 m, −25%; non-operated 0.10→0.11 m, +10%; $p>0.05$). Conclusion: the SL CMJ does not improve within a session, and the observed differences do not result from a learning effect.

C. DROP VERTICAL JUMP / DROP JUMP (DVJ / DJ)						
**Author, Year**	**n**	**Graft**	**Time (months)**	**Jump test**	**Parameter**	**Key findings and interpretation**
Andrade 2023	n=25	HT vs BPTB	6–72	DVJ	vGRF, kinematics	vGRF in ACLR patients is significantly lower than in healthy controls ($p=0.049$). No kinematic valgus differences between HT and BPTB. Conclusion: ACLR reduces loading of the operated limb in the DVJ regardless of graft type.
Hunnicutt 2024	n=24 (QT=14,PT=10)	QT vs PT	6–24	DVJ	Peak vGRF	No biomechanical DVJ differences between QT and BPTB ($p>0.05$); both groups unload the operated limb in an identical way. Conclusion: graft type QT vs BPTB does not affect landing mechanics.

Moya-Angeler 2017	n=74	HT (ST+G)	0 / 3 / 6 / 12 (preop → 12)	DVJ + VJ + SL hop	LSI (%)	Bilateral tests (DVJ, VJ): LSI <90%; single-leg tests (SL hop): LSI >90%. Conclusion: bilateral tests are more sensitive in detecting asymmetry than single-leg hops, which may falsely mask deficits.
Papalia 2014	n=40 (HT=20,BPTB=20)	HT vs BPTB	3 and 6	DVJ landing	KAM	At 6 months, BPTB combined with proprioceptive rehabilitation yields significantly better KAM values (smaller dynamic valgus) than HT ($p<0.0001$). Conclusion: BPTB + proprioceptive training reduces the risk of re-injury
Gaugg 2026a	n=26 (ST=15,QT=11)	ST vs QT	8–9	VDJ	Valgus	Women show greater contralateral knee valgus than men ($p=0.016$). No differences ST vs QT in the hop test. Conclusion: sex moderates the valgus pattern regardless of graft – the risk is greater in women.
Gaugg 2025	n=26 (ST=15,QT=11)	ST vs QT	8	VDJ	Max valgus	At 8 months, QT yields significantly smaller dynamic valgus (+3.5° varus) than ST (−2.4° valgus; $p=0.027$). Conclusion: QT harvesting potentially carries a lower risk of re-injury to the ACL than ST.
Xue 2024	n=48	Not specified	12–36	DVJ	EMG, kinematics	In ACLR patients, activation of the vastus medialis, rectus femoris and vastus lateralis muscles of the operated limb is lower than in healthy controls. Conclusion: preoperative limb dominance moderates the EMG asymmetry pattern.

Ortiz 2014	n=30 (ACLR=14,C=16)	ST+G auto	12–60 (1–5 years)	DJ 1/2-leg	Valgus, EMG	1–5 years after HT, women retain a tendency toward greater dynamic DJ valgus than healthy controls. Conclusion: women remain a high biomechanical-risk group even after multi-year rehabilitation.

D. SQUAT JUMP (SJ) AND CLASSIC SINGLE-LEG VERTICAL JUMP (VJ)						
**Author, Year**	**n**	**Graft**	**Time (months)**	**Jump test**	**Parameter**	**Key findings and interpretation**
Fontenay 2014	n=29	BPTB auto	6–9	single-leg SJ	WTotal, Hflight, LSI	At 6–9 months after BPTB, LSI Hflight = 65% vs 89% in healthy controls (p<0.001). The deficits arise mainly from the hip and ankle joints rather than the knee joint. Conclusion: compensation by adjacent joints reduces jump performance.
Pasquini 2017	n=30 (BPTB=15,HT=15)	BPTB vs HT	12	SJ (acceler.)	SJ height (cm)	At 12 months, BPTB yields higher operated-limb SJ height than HT (p=0.037). Conclusion: in the concentric test (without an eccentric phase) BPTB is more favourable than HT – the opposite direction to the CMJ – suggesting a hamstring power deficit after HT.
Jordan 2017	n=22 (ACLR=11,C=11)	Mixed (mainly ST)	39.3 ± 32.6 (3.3 years)	repeated SJ 80s	AI, EMG, height	At 3 years, concentric-phase AI in the ACLR group is significantly elevated vs healthy controls; the fatigue protocol does not worsen AI. Conclusion: SJ rebound deficits persist in the long term regardless of fatigue – a lasting change in the movement pattern.

Kurz 2022	n=17 ACLR, 67 C	HT auto	11 (6–23)	single-leg SJ + DJ	SJ power, DJ TOE	At 11 months after HT, despite clinical return to sport, SJ peak power and DJ rebound efficiency are still lower than in healthy controls. Conclusion: the time criterion (RTS) does not reflect full recovery of power.
Bach 1998	n=97	BPTB auto	60–108 (5–9 years)	single-leg VJ	S-S asym. (%)	At 5–9 years after BPTB, VJ asymmetry is <2% (LSI 98%). Conclusion: at long-term follow-up, BPTB allows full recovery of vertical-jump symmetry – the effects of patellar tendon harvesting are reversible over a few years.
Bayrakci 2008	n=37	BPTB auto	6	single-leg VJ	VJ height	At 6 months after BPTB in soccer players, VJ height is significantly lower on the operated side ($p<0.05$); quadriceps strength operated/non-operated = 75%; no correlation of VJ with isokinetic PT.
Petschnig 1998	n=50 ACLR, 16 C	BPTB auto	11.7 ± 5.9	single-leg VJ rebound	VJ height, PT corr.	At 12 months after BPTB, the single-leg VJ rebound is lower on the operated side and correlates strongly with quadriceps strength ($r=0.51$, $p<0.001$).
Gobbi 2003	n=80	ST (quadrupl.)	36 (24–52)	single-leg VJ + hop	VJ and hop	After ST (quadrupled bone), single-leg VJ height normalizes already from 6 months, and full isokinetic strength from 12 months. Conclusion: the quadrupled-bone-ST technique offers a faster recovery path than classic BPTB – an alternative for athletes.

Sun 2009	n=156	BPTB auto vs allo	67,2 (56–102; 5.6 years)	VJ Harner + hop	LSI VJ	At 5.6 years, no VJ differences between BPTB autograft and allograft (p>0.05). Conclusion: allograft is a viable functional alternative to autograft in the long-term vertical-jump perspective – there is no basis for preferring one over the other.
Sun 2011a	n=69	HT allo ir vs non-ir	41	VJ Harner + hop	LSI VJ	At 3.5 years, irradiation of the HT allograft does not affect VJ height, even though it worsens knee joint stability. Conclusion: joint stability and jump function are independent domains – irradiation selectively affects the former.
Sun 2011b	n=166	HT auto vs allo	67 (5.6 years)	VJ Harner + hop	LSI VJ	At 5.6 years, no VJ differences between HT autograft and fresh-frozen allograft (p>0.05). Conclusion: no preference between HT types – graft choice may rest on criteria other than jump performance.
Tian 2016	n=87	HT auto vs ir-allo	59 (5 years)	VJ Harner + hop	LSI VJ	At 5 years with the double-bundle technique, VJ height does not differ between HT autograft and irradiated allograft (p=0.677). Conclusion: the DB technique eliminates differences between HT graft types with respect to jump performance.
Fox 2004	n=32 revision	BPTB allo	57,6 (25–145)	single-leg VJ	S-S asym.	In revision ACLR (BPTB allograft), mean VJ symmetry = 98%, but variability is very high (SD 33.2%; range 35–232%). Conclusion: revision allografts yield unpredictable functional results – they require individual assessment of each patient.

Oeffinger 2000	n=23	BPTB auto vs allo	auto: 27.6 ± 10<br>allo: 21.3 ± 5	single-leg VJ	VJ height (cm)	At 25 months, no VJ differences between BPTB autograft and allograft (p=0.47); both groups within the normal range relative to controls. Conclusion: the type of BPTB (auto/allo) does not affect vertical-jump function, but delayed gastrocnemius EMG latencies persist in both groups.

LEGEND:

GRAFTS:

BPTB – Bone-Patellar Tendon-Bone (bone–patellar tendon–bone)

HT – Hamstring Tendon (hamstring muscle tendons: semitendinosus ± gracilis)

ST – Semitendinosus (semitendinosus muscle tendon)

QT – Quadriceps Tendon (quadriceps femoris tendon)

STGR – Semitendinosus + Gracilis (semitendinosus and gracilis muscle tendons)

LET – Lateral Extra-articular Tenodesis

auto / allo – autograft / allograft

ir / non-ir – irradiated / non-irradiated; DB – double-bundle

VERTICAL-JUMP TESTS:

CMJ – Countermovement Jump

SJ – Squat Jump (from a squat, without countermovement)

DVJ / VDJ – Drop Vertical Jump (vertical jump following a drop from a raised platform)

DJ – Drop Jump (drop landing with immediate rebound)

SL CMJ / SL VJ – Single-Leg CMJ / Vertical Jump (single-leg vertical jump)

VJ – Vertical Jump; VJ Harner – jump test according to the Harner protocol

BIA – Back in Action (standardized battery of functional tests)

FP – Force Plate; dual FP – dual force plates

OUTCOME PARAMETERS:

LSI – Limb Symmetry Index = (operated / non-operated limb) × 100%

AI – Asymmetry Index (inter-limb asymmetry index)

RSI – Reactive Strength Index (jump height / ground contact time)

RSImod – Modified RSI (jump height / contraction time)

vGRF – Vertical Ground Reaction Force

PP – Peak Power (peak mechanical power)

RFD / RFDe – Rate of Force Development / eccentric

KAM – Knee Abduction Moment (knee abduction/valgus moment)

SSC – Stretch-Shortening Cycle

JH – Jump Height; TTS – Time to Stability

TOE – Take-off Efficiency; WTotal – total mechanical joint work

Hflight – flight-phase height; S-S – side-to-side

ecc – eccentric phase; con – concentric phase

OTHER ABBREVIATIONS:

ACLR – anterior cruciate ligament reconstruction

EMG – electromyography
